# Enrichment of periodontal pathogens from the biofilms of healthy adults

**DOI:** 10.1038/s41598-019-41882-y

**Published:** 2019-04-02

**Authors:** Monika Naginyte, Thuy Do, Josephine Meade, Deirdre Ann Devine, Philip David Marsh

**Affiliations:** Division of Oral Biology, School of Dentistry, University of Leeds, Leeds, UK

## Abstract

Periodontitis is associated with shifts in the balance of the subgingival microbiome. Many species that predominate in disease have not been isolated from healthy sites, raising questions as to the origin of these putative pathogens. The study aim was to determine whether periodontal pathogens could be enriched from pooled saliva, plaque and tongue samples from dentally-healthy adult volunteers using growth media that simulate nutritional aspects of the inflamed subgingival environment. The microbiome was characterised before and after enrichment using established metagenomic approaches, and the data analysed bioinformatically to identify major functional changes. After three weeks, there was a shift from an inoculum in which *Streptococcus*, *Haemophilus*, *Neisseria*, *Veillonella* and *Prevotella* species predominated to biofilms comprising an increased abundance of taxa implicated in periodontitis, including *Porphyromonas gingivalis*, *Fretibacterium fastidiosum*, *Filifactor alocis*, *Tannerella forsythia*, and several *Peptostreptococcus* and *Treponema* spp., with concomitant decreases in health-associated species. Sixty-four species were present after enrichment that were undetectable in the inoculum, including *Jonquetella anthropi*, *Desulfovibrio desulfuricans* and *Dialister invisus*. These studies support the Ecological Plaque Hypothesis, providing evidence that putative periodontopathogens are present in health at low levels, but changes to the subgingival nutritional environment increase their competitiveness and drive deleterious changes to biofilm composition.

## Introduction

The mouth harbours a diverse and natural microbiota that persists on oral surfaces as structurally- and functionally-organised multi-species biofilms that have a symbiotic relationship with the host^1,2^. The host provides a warm and nutritious habitat, while the resident oral microbiota delivers important health benefits (e.g. pathogen exclusion, immune modulation, entero-salivary nitrate reduction cycle)^3,4^.

A dynamic balance exists between the host and the oral microbiota, and substantial changes in the local environment can drive deleterious shifts in the microbial composition of dental biofilms, and these can predispose a site to disease (dysbiosis). For example, the frequent intake of fermentable dietary sugars and/or reductions in saliva flow result in dental biofilms experiencing extended periods of low pH. This selects for acidogenic/acid-tolerating species at the expense of beneficial oral bacteria that preferentially grow at neutral pH^5,6^, and increases the risk of dental caries. In contrast, gingivitis and periodontitis are associated with an inflammatory response to excessive biofilm accumulation around the gingival margin. This response can be de-regulated and subverted by some bacterial populations leading to a heightened expression of pro- inflammatory molecules, and an increased flow of gingival crevicular fluid (GCF; a protein-rich serum-like exudate). Metagenomic studies have shown that the microbiome in periodontal pockets is markedly different from that found in health, and contains high proportions of obligately anaerobic, often proteolytic taxa, some of which have yet to be grown in the laboratory while other have yet to be named^7–9^.

Although some periodontal pathogens have been detected on occasions, and at low levels, in samples from periodontally-healthy individuals^10–12^, many of the organisms that have been more recently implicated in disease^7^ have only been detected at inflamed sites. The factors that drive the changes in the microbiota in periodontal disease are not fully understood, and a number of theories have been postulated to explain the shift from a symbiotic to a dysbiotic relationship with the host. These theories range from exogenous infection^13^, co-infection with viruses^14^, enrichment of minor species within the biofilm following changes to the local environment^15^ through to low abundance keystone pathogens orchestrating commensal species to provoke a destructive inflammatory response^16^, but experimental evidence for these concepts is sparse. The aim of the present study was to investigate whether any of the recently described putative periodontal pathogens^7^ could be enriched from biofilms taken from dentally-healthy young adult volunteers by growth under nutritional conditions that reflect aspects of the subgingival environment found during inflammation. Samples of saliva, tongue and supragingival plaque from the volunteers were pooled to increase the probability of including putative periodontal pathogens in the inoculum prior to enrichment.

## Results

**Figure 1:**
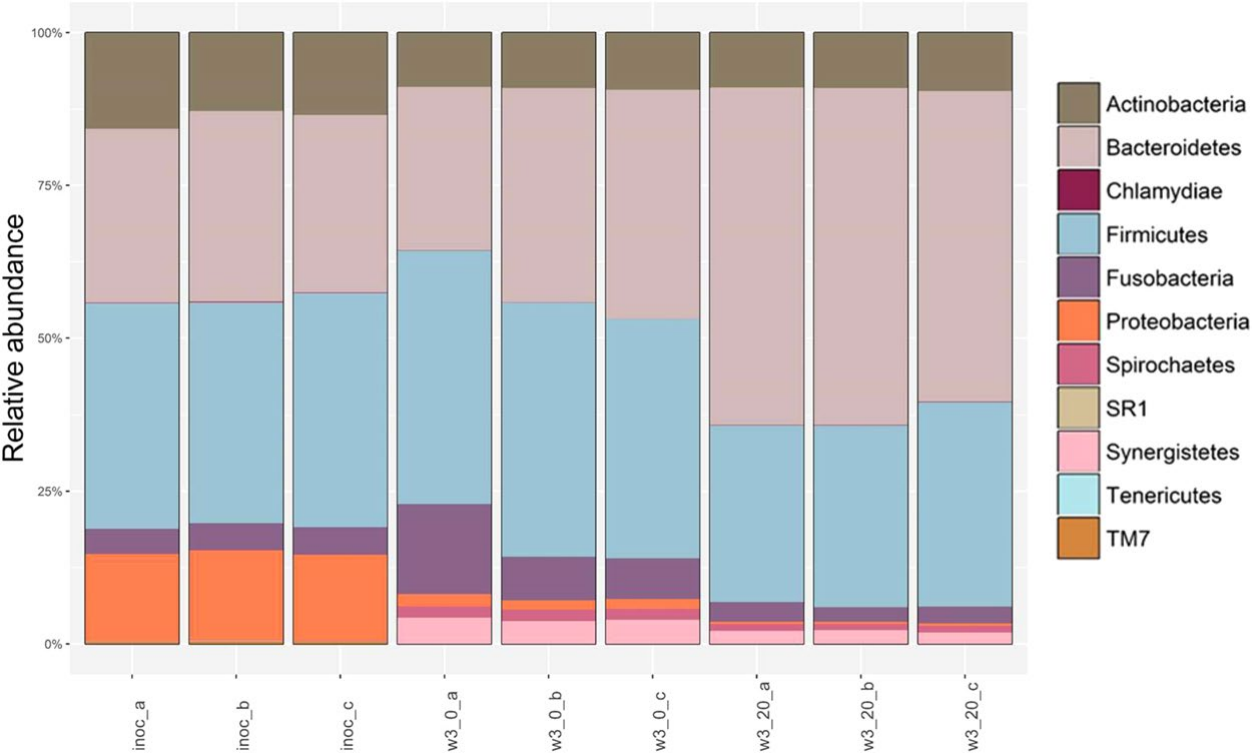
The taxonomic structure of the samples before and after enrichment. Relative phyla distribution of the inoculum and of the biofilms following enrichment are shown. Analysis based on the shotgun metagenomic data shows the differences in relative phyla distribution between inoculum and biofilms enriched in a protein-rich medium ± serum. The corresponding 16S rRNA data were used to construct a similar phyla distribution, which is displayed in Supplementary Fig. 2. Inoc – inoculum sample, w3_0 – biofilms cultured in protein-rich medium, w3_20 – biofilm cultured in protein-rich medium with 20% serum (v/v), a, b, c – independent replicates.

Pooled oral bacterial samples from eight volunteers were subjected to enrichment culture in two types of medium (protein-rich with or without supplementation with serum) for three weeks, and the microbial composition and potential functions were compared between biofilms enriched on these different media and between the biofilms and the inoculum. Three independent enrichment cultures were performed under each set of nutritional conditions, and the reproducibility of the resultant microbial communities can be gauged from the similarity of the profiles of the taxa in Fig. 1. On average, 22.4 million sequences were obtained per sample (range 20.7–26 million). MEGAN was used for the analysis of taxonomy and functional potential content of our biofilm samples. Analysis of metagenomes showed that the sequence reads represented 304 OTUs at species level and 4490 functionally annotated genes of bacterial origin (Supplementary Dataset and Table 1). Only 28–35.5% of reads were assigned a functional role.

**Table 1:** Summary of fold increase in relative abundance of detected species in inoculum and biofilms enriched in protein-rich medium (PRM) with and without supplementation with serum.

Enriched in PRM		Enriched in PRM + serum	
Species	Fold change (log2)	Species	Fold change (log2)
**Comparison with inoculum**			
*Eggerthia catenaformis*	19.5***	*Pseudoramibacter alactolyticus*	18.3***
*Anaeroglobus geminatus*	19.1***	*Eggerthia catenaformis*	17.7***
*Eubacterium nodatum*	17.5***	*Olsenella uli*	17.3***
*Treponema denticola*	16.6***	*Anaerolineaceae* sp. OT 439	16.9***
*Bacteroidetes* sp. OT 272	15.6***	*Anaeroglobus geminatus*	16.4***
*Treponema maltophilum*	15.5***	*Eubacterium nodatum*	15***
*Prevotella oralis*	15.4***	*Peptostreptococcaceae* sp. OT 113	14.9***
*Streptococcus constellatus*	14.8***	*Eubacterium yurii*	14.8***
*Prevotella marshii*	14.5***	*Mogibacterium* sp. CM50	14.3***
*Pseudoramibacter alactolyticus*	14.5***	*Treponema maltophilum*	14***
**Comparison between biofilms enriched on different growth media**			
*Prevotella saccharolytica*	12.4***	*Actinomyces cardiffensis*	11.8***
*Prevotella oulorum*	12.2***	*Porphyromonas gulae*	11.6***
*Campylobacter showae*	11.8***	*Prevotella intermedia*	10.8***
*Desulfovibrio desulfuricans*	11.3***	*Prevotella dentalis*	10.4***
*Prevotella veroralis*	11***	*Porphyromonas gingivalis*	4.3***
*Oribacterium parvum*	10.9***	*Olsenella uli*	4***
*Megasphaera micronuciformis*	10.8***	*Pseudoramibacter alactolyticus*	3.9***
*Streptococcus oralis*	10.8***	*Anaerolineaceae bacterium* OT 439	3.6***
*Prevotella* sp. OT 317	10.7***	*Alloprevotella tannerae*	1.9***
*Campylobacter rectus*	10.6***	*Peptostreptococcaceae* sp. OT 113	1.7*

The experimental design was set to compare the relative abundance of OTUs in the inoculum to abundance in the PRM ± serum groups. The ten taxa that had the greatest fold increase are listed for each enrichment condition. *p < 0.05, **p < 0.01, ***p < 0.001, Wald test.

### Microbial composition of the inoculum

**Figure 2:**
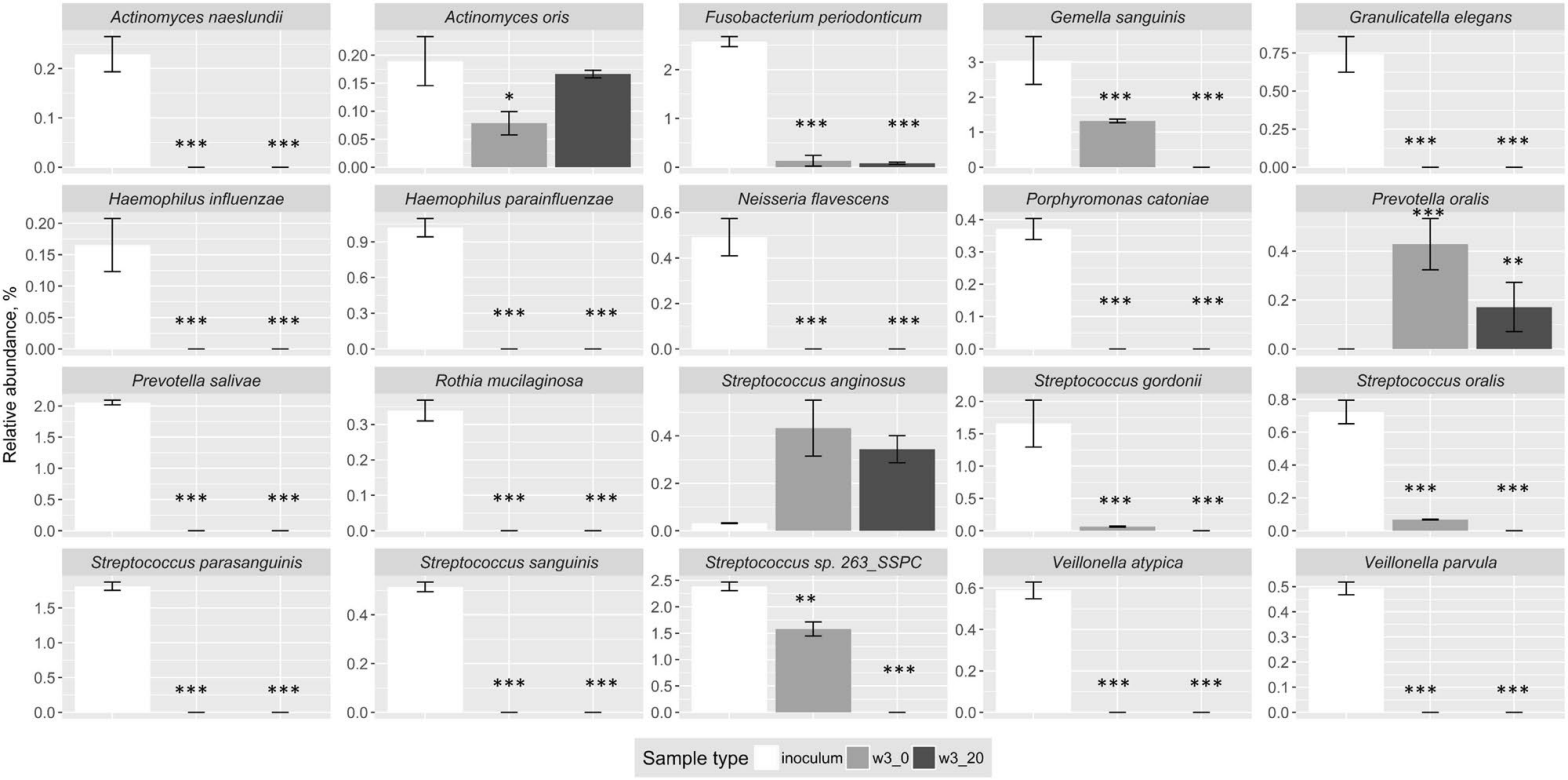
Decrease in relative abundance of selected bacterial species associated with oral health following enrichment of saliva and biofilms pooled from healthy adults in a protein-rich medium with or without supplementation with serum. Graphs summarise the changes in relative abundance of metagenomes rarefied to the same sequencing depth between inoculum and biofilms following three weeks enrichment on a protein-rich medium with or without serum. Inoculum – inoculum sample, w3_0 – biofilms cultured in protein-rich medium, w3_20 – biofilm cultured in protein-rich medium with 20% serum (v/v). Asterisks mark the significant differences in relative abundance compared to the inoculum, *p < 0.05, ***p < 0.001, HSD test.

**Figure 3:**
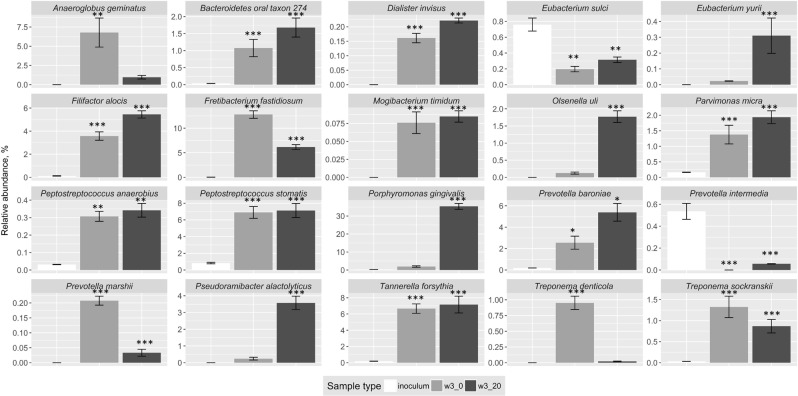
Difference in relative abundance of selected species implicated in periodontal disease following enrichment of saliva and biofilms pooled from healthy adults in a protein-rich medium with or without supplementation with serum. Graphs summarise the changes in relative abundance of metagenomes rarefied to the same sequencing depth between inoculum and biofilms following three weeks enrichment in a protein-rich medium with or without serum. Other changes are listed in Table 1. Inoculum – inoculum sample, w3_0 – biofilms cultured in protein-rich medium, w3_20 – biofilm cultured in protein-rich medium with 20% serum (v/v). Asterisks mark the significant differences in relative abundance compared to the inoculum, *p < 0.05, **p < 0.01, ***p < 0.001, HSD test. Selection of the species shown is based in part on the evidence of their association with periodontal disease as described by Perez-Chaparro *et al*.^7^.

At the phylum level, the inoculum (comprising pooled samples of tongue biofilm, supragingival molar plaque and saliva from healthy volunteers) was rich in Firmicutes, Bacteroidetes and Proteobacteria, and contained high proportions of the following genera: *Streptococcus*, *Haemophilus*, *Veillonella*, *Neisseria* and *Prevotella* (Fig. 1 and Table 2). Predominant species included *Streptococcus sanguinis*, *Streptococcus salivarius*, *Streptococcus cristatus* and *Haemophilus parainfluenzae* (Fig. 2). In contrast, members of genera associated with periodontal disease, such as *Porphyromona*s, *Filifactor*, *Tannerella*, and *Treponema*, were either not detected in the inoculum, or were present at very low levels using shotgun metagenomics. At the species level, many of the taxa that have recently been implicated with periodontal disease^7^, including members of the ‘red complex’ (*Porphyromonas gingivalis*, *Treponema denticola*, *Tannerella forsythia*), were detected in low abundance in the pooled inoculum at the start of the enrichment studies (see Fig. 3, Supplementary Table 1, and Supplementary Fig. 2).

**Table 2:** Distribution of the most abundant genera in the inoculum and biofilm samples.

Genus	Inoculum (%)	Protein-rich medium (%)	Protein-rich medium + serum (%)
*Bacteroides*	0	0.1	0.1
*Tannerella*	1–1.1	6.4–7.5	6.7–8.6*
*Alloprevotella*	7.8–8.8	0.2–0.3*	0.4–0.5
*Prevotella*	19.2–20.8	3.7–4.9*	4.8–6.5
*Capnocytophaga*	0.6–0.8	0.1	0
*Fusobacterium*	3.3–3.4	1.7–3.4	0.5–0.7*
*Neisseria*	1.5–2.4	0	0
*Haemophilus*	1.6–1.9	0	0
*Treponema*	0.1	2.5–2.9*	0.9–1.2
*Fretibacterium*	0.1	12–13.5*	5.6–6.5
*Actinomyces*	5.8–8	0.1*	0.3–0.4
*Slackia*	0	0.2–0.4	0.3–0.4
*Granulicatella*	1.3–1.4	0	0
*Streptococcus*	10.6–11.7	1.03–1.26	0.5–1.1*
*Mogibacterium*	1.1–1.4	2.3–3.3	1.2–2.4
*Peptostreptococcus*	0.8–1.1	15.1–16.8*	8.8–12
*Anaeroglobus*	0	4.6–8.2*	0.8–1.2
*Dialister*	0	0.1–0.2	0.2
*Veillonella*	3.6–4	0	0
*Parvimonas*	0.2	1.1–1.8	1.9–2.3*
*Porphyromonas*	4.3–4.8	1.9–2.8	34–37.1*
Other	31.3–33.7	38.3–41.2	24.6–28.6*

Samples of the inoculum were compared with biofilms cultured in PRM with and without serum; *p < 0.05, two-tailed multiple comparison after Kruskal-Wallis.

### Diversity of biofilms following enrichment

**Figure 4:**
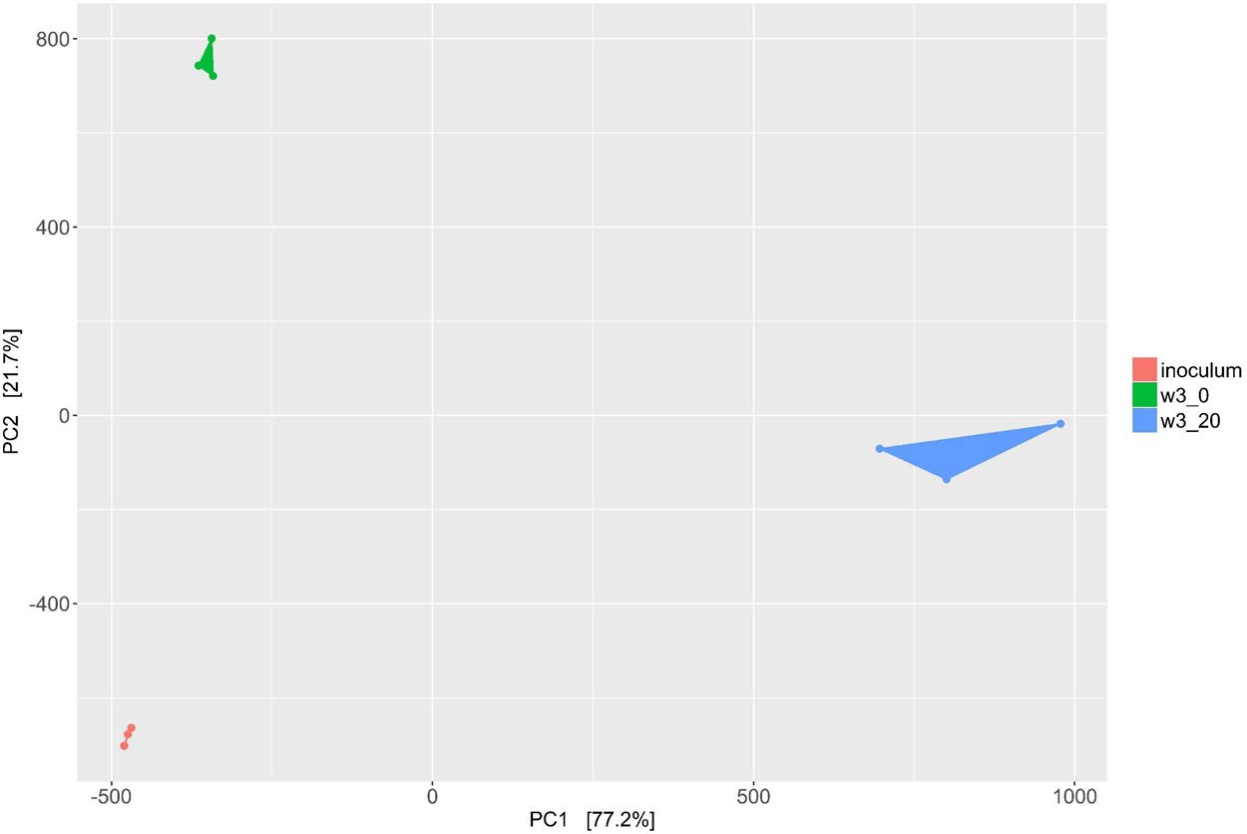
Principal component analysis displaying the difference in relation between samples. Redundancy analysis and Bray-Curtis distance index methods were used to plot samples. Inoculum – inoculum samples, no_serum – biofilms cultured in protein-rich mediums, serum (w3_0)– samples cultured in protein-rich medium with 20% serum (v/v) (w3_20).

The number of species (alpha diversity) was significantly lower in cultured biofilms than in the inoculum (p < 0.05, Kruskal-Wallis). There was a significant decline of unique species in biofilms but there were no differences in alpha diversity between enrichments performed in media with or without serum (Supplementary Fig. 2). Shannon index revealed higher diversity and evenness of the inoculum compared with enriched biofilms, p < 0.05, with no significant differences between biofilm samples, irrespective of the growth medium. Redundancy analysis revealed that samples were similar between experimental replicates and distinct between experimental comparison groups (inoculum and biofilms cultured in two distinct media) (Fig. 4).

### Composition of biofilms following enrichment

There was a shift in the distribution of phyla following enrichment and biofilm growth. Biofilm samples had lower proportions of Actinobacteria, Proteobacteria, TM7 and SR1 phyla than the inoculum, while there were increases in Bacteroidetes, Synergistetes and Firmicutes following enrichment (Fig. 1). Bacteria associated with gingival health, especially those belonging to the genera *Streptococcus*, *Granulicatella*, *Rothia*, *Haemophilus* and *Veillonella*, decreased in relative abundance following enrichment and biofilm growth. Many species present in the inoculum, and which are associated with oral health, were not detected in biofilms or showed a low relative abundance (Fig. 2). For example, *H*. *parainfluenzae*, and *Veillonella* and several *Streptococcus* spp. (e.g. *S*. *gordonii*, *S*. *oralis*, *S*. *sanguinis*, *S*. *parasanguinis*) were prevalent in the inoculum but were not detected in biofilms following enrichment for three weeks.

In contrast, 64 species were found in biofilms that could not be detected in the inoculum (Supplementary Table 1). Many of these species are fastidious, and included *Jonquetella anthropi*, *Desulfovibrio desulfuricans*, *Dialister invisus*, *Treponema maltophilum* and *Prevotella marshii*. The nutrient status of the enrichment medium had an impact on the composition of the developing biofilms (Fig. 3, Table 1). Compared with the inoculum, biofilms grown in a protein-rich medium lacking serum were enriched with Synergistetes, Spirochetes and Bacteroidetes, (p < 0.05, Kruskal-Wallis), whereas just higher proportions of Bacteroidetes were found following growth with serum (Fig. 1). Biofilms were dominated by species associated with periodontal disease after growth in either medium (Fig. 3; Table 1). Some putative pathogens were enriched preferentially in protein-rich medium (PRM) alone (e.g. *Fretibacterium fastidiosum*, *Prevotella marshii*) while others were enhanced in the presence of serum (e.g. *Filifactor alocis*, *Pseudoramibacter alactolyticus*) (Fig. 3, Table 1, Supplementary Table 1). Some *Fusobacterium* species (e.g. *F*. *periodonticum*) were more abundant in biofilms cultured in protein-rich medium (Fig. 2). However, in general, the *Fusobacterium* genus was most abundant in inoculum samples and was not favoured by culturing in either medium, p > 0.05 (Table 2). The species that make up the ‘red complex’ showed differential responses to medium supplementation. *Tannerella forsythia* was enriched by the protein-rich medium irrespective of whether it was supplemented with serum, while *P*. *gingivalis* and *T*. *denticola* were more strongly favoured by PRM with serum or PRM alone, respectively (Fig. 3). The responses of *P*. *gingivalis* in the enrichment studies, as determined by the metagenomic approach, were confirmed by use of qPCR (Supplementary Fig. 3).

### Functional analysis of enriched biofilms

The inoculum was over-represented with genes involved in di- and oligo-saccharide metabolism, such as sucrose, fructo-oligosaccharide and raffinose utilization, lactose and galactose uptake, haem uptake in Gram-positive bacteria, oxidative stress, urea degradation or sialic acid metabolism (Supplementary Tables 2–4).

In contrast, biofilm samples following enrichment culture were over-represented with genes implicated in virulence (resistance to antibiotics, conjugative transposons, ABC transporters, multidrug resistance efflux pumps), amino acid metabolism (including for branched amino acids), anaerobic respiratory reduction, and cobalamin synthesis and metabolism (Supplementary Dataset file). Samples that were cultured in the medium with serum had more over-represented functional groups related to virulence and antibiotic resistance compared with biofilms cultured without serum.

## Discussion

The oral microbiome exists as complex multi-species biofilms on oral surfaces, especially on teeth, and oral hygiene can maintain dental plaque at levels compatible with health. Indeed, the oral microbiome has a symbiotic relationship with the host, and delivers important health benefits. However, this relationship can breakdown as a result of changes to the composition and metabolism of the microbiota at a site. In periodontal disease, there are substantial increases in the proportions of obligately anaerobic and proteolytic bacteria, many of which are Gram negative. The source of some of these bacteria is unclear, as many have not been detected at healthy sites, while the drivers of these dysbiotic changes in the microbiome are not fully understood.

In the healthy mouth, the microbial composition of oral biofilms can remain relatively stable over time, despite regular but minor perturbations to the environment^17,18^. Although there are inter-subject variations in the microbiota, attempts have been made to define a core microbiome associated with oral health and, depending on the study design, this includes representatives of the genera *Streptococcus*, *Veillonella*, *Granulicatella*, *Neisseria*, *Haemophilus*, *Corynebacterium*, *Rothia*, *Actinomyces*, *Prevotella* and *Fusobacterium*^19–21^. The inoculum for the enrichment studies described here was obtained by pooling samples from saliva, tongue and supragingival plaque from healthy volunteers. Samples were pooled to increase the probability that some of the putative pathogens that have been associated with periodontal disease^7^ might be present in the inoculum. Many of these species have not been reported in health and, therefore, may not have been present in a site-specific sample from a single individual. The in-depth characterisation of the inoculum using contemporary metagenomic approaches confirmed that it was comprised of genera representative of the core oral microbiome listed above, and included many species associated with oral health such as *S*. *oralis*, *S*. *sanguinis*, *S*. *gordonii*, *Granulicatella elegans*, *Neisseria flavescens*, and *Porphyromonas catoniae*.

As described above, the symbiotic relationship between the host and the oral microbiome can break down and disease can occur. The inflammatory response to plaque accumulation that occurs in periodontal disease ensures the rapid delivery of an array of host defence factors to counter the microbial insult; however, GCF also contains proteins and glycoproteins that can be exploited as nutrients by many of the fastidious, proteolytic and ‘inflammophilic’ bacteria associated with these pro-inflammatory biofilms^22^.

The source of these fastidious micro-organisms, some of which cannot be detected in health, and the drivers that enable them to become predominant remain to be elucidated. One line of reasoning has been that changes to the subgingival environment associated with inflammation, especially in terms of nutrient profile, select for micro-organisms most adapted to these altered conditions. Early studies showed that repeated enrichment of subgingival biofilms from patients with periodontitis in human serum led to the eventual selection of *Bacteroides intermedius* (now classified as *Prevotella intermedia*) from samples in which this species was not originally detected^23^. Subsequent enrichment studies using continuous culture in the same serum-based medium generated consortia capable of degrading molecules involved in host defence (e.g. immunoglobulins, complement, haptoglobin, transferrin, etc), and spirochaetes and obligately anaerobic Gram-negative bacteria predominated^24^. In these studies, however, the plaque samples used for the inoculum were from moderately deep and untreated periodontal pockets, and neither the inoculum nor the resultant communities were grown as biofilms nor were characterised in detail. We, therefore, used an inoculum comprising saliva, supragingival plaque and tongue scrapings from dentally-healthy young adults, and grew them on surfaces for three weeks, with regular changes of the medium, and characterised the inoculum and enriched biofilms using contemporary metagenomic approaches. Previous studies had incubated saliva samples in the CBD for two weeks in a proof-of-principal study to demonstrate the utility of this model to grow complex communities of oral bacteria^25^. We used a slightly longer incubation period to provide the maximum opportunity for the enrichment of slow-growing and nutritionally-fastidious species to outcompete the health-associated bacteria that predominated in the inoculum taken from dentally-healthy subjects. In our study, the culturing conditions reflected some aspects of an inflamed periodontal pocket, such as an anaerobic atmosphere and protein-rich environment, etc. Species that benefited from this environment eventually outcompeted the facultatively anaerobic species that were abundant in the inoculum (e.g. *Streptococcus* and *Neisseria* species). When analysed at the genus and species level, there were marked differences between the inoculum and the biofilms, and between the biofilms that were cultured with and without serum. Several species present in the inoculum, and associated with health, were non-competitive during enrichment, and some were not detected after three weeks (e.g. *H*. *parainfluenzae*, *S*. *sanguinis*, *S*. *gordonii*, *N*. *flavescens*). Other species thrived under the altered nutritional conditions, and their relative abundance increased markedly. Many of these species have been isolated from inflamed periodontal pockets and are associated with tissue destruction, and included the bacteria known as the ‘red complex’ (*P*. *gingivalis*, *T*. *denticola*, *T*. *forsythia*). The medium used for the enrichment experiment influenced the composition of the resultant microbial communities. Some species were more abundant in the absence of serum (e.g. *F*. *fastidiosum*, *Anaeroglobus geminatus*, *P*. *marshii* and *T*. *denticola*) while others (including *P*. *gingivalis*, *F. alocis*, *P*. *alactolyticus* and *E*. *yurii*) benefited more from serum supplementation, demonstrating that the enriched organisms could exploit a wide repertoire of proteins.

It was noteworthy that 64 species detected in the biofilms following enrichment could not be found in the inoculum, even when using a metagenomic approach. Many of these organisms have fastidious growth requirements (e.g. sulphate-reducing species, spirochaetes). As this was a closed system, these findings demonstrate that these taxa were present in biofilms from healthy mouths, but must be non-competitive in their growth relative to health-associated species, and were present below the level of detection. However, once environmental conditions were changed and became more favourable, then they were able to exploit the altered nutritional profile and out-compete many species associated with health, and become predominant members of the community. Likewise, many species associated with oral health became non-detectable, or were present in very low abundance, following enrichment culture.

The application of metagenomics approaches to characterise the oral microbiome has resulted in a diverse collection of new or previously unknown organisms being associated with periodontitis, although their precise roles in disease are unknown. A systematic review of 41 studies evaluated the strength of evidence linking these newly identified pathogens with periodontitis^7^. In our study, seven of 17 taxa with ‘moderate evidence’ and three of 15 taxa with ‘some evidence’ for an association with periodontitis, had a higher abundance in the enriched biofilms compared with the inoculum.

Functional potential analysis showed clear differences between the inoculum and biofilms cultured in media with or without serum. The inoculum was over-represented with genes responsible for carbohydrate metabolism, including di- and oligo-saccharide utilization, while the enriched biofilms had an abundance of genes associated with proteolysis, methanogenesis, virulence, motility and chemotaxis (Supplementary Tables 3 and 4). These findings are consistent with results from functional studies of biofilms taken from healthy individuals and from patients with generalised chronic periodontitis^8^.

These findings support the concepts behind the original ‘ecological plaque hypothesis’^15,26^ and the more recent ‘polymicrobial synergy and dysbiosis’ model of periodontal disease^27^, in which the microbiota isolated from pockets gains an advantage from the substrates derived from inflammation and tissue breakdown, and this drives community restructuring. Disease is a consequence of a dysbiotic shift in the microbiota driven by a change in the local environment. Implicit in these concepts is that disease can be managed or prevented by interfering with the drivers of dysbiosis. When the inflammatory environment was controlled in a *P*. *gingivalis*-induced model of periodontitis in rabbits using Resolvin E1, there was tissue regeneration and a decrease in Gram-negative anaerobic species^28^.

In the present study, samples of saliva and biofilm from teeth and tongue from healthy volunteers were pooled to create the inoculum. This was because we could not predict whether the enrichment cultures would be successful, and we wanted to maximise the probability of detecting putative periodontal pathogens. Now we have succeeded in developing a model of ‘pathogen enrichment’, this approach could be applied to individual samples of biofilm to see whether these organisms are present at all sites and in all individuals, or whether their distribution is highly localised or they colonise only a subset of people. If the latter was the case then it might be possible to identify individuals at risk of periodontitis in advance of the development of disease and focus remedial therapy on this group.

## Materials and Methods

### Sample collection

Eight dentally-healthy volunteers (mean age 31 ± 8 y, 50% male: 50% female) provided samples of supragingival buccal molar plaque, biofilm from the tongue dorsum and 5 ml of unstimulated saliva. Supragingival plaque and tongue biofilm samples were each collected using a sterile wooden toothpick and a sterile wooden spatula, respectively, and placed into 3 mL sterile pre-reduced protein-rich medium and transferred to an anaerobic workstation (Don Whitley Scientific; Shipley) together with the saliva samples within 60 minutes. Each sample was homogenised by vortexing for 60 seconds, and then pooled to obtain separate saliva, tongue and supragingival plaque samples, and vortexed for an additional 60 seconds. Inclusion criteria were that participants routinely brushed their teeth twice daily, attended regular dental check-ups, and were not undergoing any treatment for dental caries or periodontal disease, nor had been on antibiotics for at least three months or were having treatment for systemic disease. Moreover, as part of the inclusion criteria, participants were asked to confirm that they were not undergoing dental treatment and did not have overt periodontitis or caries disease.

### Ethics statement

Informed consent was obtained from all participants. Ethics approval was granted by the University of Leeds Dental Research Ethics Committee (020915/MN/175). All sample processing was carried out in accordance with the relevant guidelines and regulations.

### Enrichment cultures

Enrichment cultures were performed using the Calgary Biofilm Device (CBD; Innovotech, Edmonton, Canada)^29^. This device is a 96-well microtitre plate with a modified lid containing pegs that protrude into the growth medium in each well, enabling biofilm formation on the pegs. The hydroxyapatite-coated pegs were preconditioned with 200 μL sterile human saliva^30^ from a single 28 year old healthy female donor. The sterility of saliva was checked by aerobic and anaerobic culture for 72 h. Each well was filled with 91 μL pooled saliva and 54.5 μL of pooled supragingival plaque and 54.5 µL of pooled tongue biofilms, to contain 200 μL inoculum per well. A protein-rich medium was used to simulate the subgingival environment, and consisted of (g/L): proteose peptone (2.0), tryptose peptone (1.0), yeast extract (1.0), cysteine (0.1), porcine gastric mucin (2.0), NaCl (3.04), KCl (1.39), ascorbic acid (0.0016), KH_2_PO_4_ (0.59) and urea (5 mM), L-arginine (9 mM). In half of the wells, this medium was supplemented with 20% (v/v) heat-inactivated foetal bovine serum. All chemicals were obtained from Sigma-Aldrich unless otherwise stated.

Biofilm experiments were carried out in triplicate, i.e. three individual CBD plates were used. After inoculation, the plates were incubated anaerobically in 10% H_2_, 10% CO_2_, 80% N_2_ at 37 °C in an anaerobic workstation. The medium in each well was changed after 24 h and subsequently twice a week. After three weeks of incubation, biofilms were harvested by snipping pegs from the lid with sterile pliers and scraping the biofilms carefully with a dental scaler into 500 μL of sterile phosphate buffered saline^25^. Three pegs from one CBD plate were pooled together to provide one sample from each plate. Therefore, three independent replicate samples were obtained for each condition (PRM and PRM + 20% serum). Samples were treated with 1.5 µL propidium monoazide (final concentration 50 μM) prior to DNA isolation according to the manufacturer’s instructions (Biotium, Fremont, CA) to obtain DNA only from intact cells^31^. The quantity of DNA was assessed using the Pico Green Kit (Molecular probes, Eugene, OR).

### Sequencing of metagenomes

DNA was isolated from the inoculum and also from three week biofilm samples using the UltraClean® DNA Isolation Kit according to manufacturer’s instructions (Mo Bio, Carlsbad, CA). DNA was fragmented with the Covaris system (Covaris, Woburn, MA) to obtain 200 bp DNA fragments. After quality screening, libraries were prepared with NEBNext Ultra DNA Library Prep Kit for Illumina® (New England BioLabs, Ipswich, MA) according to the manufacturer’s instructions. PCR enrichment was performed using six cycles of PCR of denaturation, annealing and extension. After assessing the quality of the libraries, 100 ng of each library were pooled and submitted for 150 bp paired-end sequencing on an Illumina HiSeq3000 (Illumina, San Diego, CA).

### Data analysis

Adapters were removed with cutadapt^32^, and sickle v.1.33 was used to quality-trim the paired-end reads. The quality threshold was set at 28, length threshold at 15. Paired-end reads were mapped against the non-redundant bacterial protein database (downloaded November 2017 from NCBI) using default parameters in diamond^33^. The metagenomes were uploaded to The MEtaGenome ANalizer (MEGAN) (v.6.8.9)^34^ for the analysis of taxonomy and functional potential using the recommended parameters (*min-score* threshold was set to 80 and *top-percent* filter was set to 10%, min support 0.01%). MEGAN was used to perform taxonomic binning across taxonomic ranks using the lowest common ancestor algorithm. Phyloseq^35^ and vegan^36^ packages in R were used to analyse alpha diversity of samples and graphically display data using ggplot2 package^37^. The DESeq2^38^ package was used for differential analysis of taxonomy and functional potential between different sample groups.

### Statistical analyses

The Tukey honest significant difference test was applied to investigate significant changes in the relative abundance between the inoculum and biofilm groups. Phyloseq and DESeq2 packages were used to test and plot the presence of differentially abundant species. The negative binomial generalised linear model procedure with Wald statistics was used with significance of p adjusted values < 0.05.

To investigate differences in functional potential between groups, data generated by MEGAN were evaluated with DESeq2 package (p adjusted < 0.05).

## Supplementary information

Supplementary Information

Supplementary Dataset 1

## Data Availability

Samples were uploaded to the MG-RAST server^39^ and are publicly available with the following accessions: mgp19402 and mgp19415.
