# Supplementary material for: Enrichment of periodontal pathogens from the biofilms of healthy adults
Source: Sci Rep. 2019 Apr 2;9:5491. doi: 10.1038/s41598-019-41882-y (PMC6445289; doi:10.1038/s41598-019-41882-y)
Supplement: Supplementary file 1 — Supplementary Information [file 41598_2019_41882_MOESM1_ESM.docx]

# Enrichment of periodontal pathogens from the biofilms of healthy adults

Monika Naginyte, Thuy Do*, Josephine Meade, Deirdre Ann Devine and Philip David Marsh

Division of Oral Biology, School of Dentistry, University of Leeds, Leeds, UK

**Running title:** Enrichment of periodontal pathogens

***Corresponding author: Dr Thuy Do**, Division of Oral Biology, School of Dentistry, University of Leeds, Leeds, UK, +44 (0)113 343 8936, [N.T.Do@leeds.ac.uk](mailto:N.T.Do@leeds.ac.uk)

**Authors contact information: Monika Naginyte**, Division of Oral Biology, School of Dentistry, University of Leeds, Leeds, UK, [naginyte@gmail.com](mailto:naginyte@gmail.com)

**Dr Josephine Meade**, Division of Oral Biology, School of Dentistry, University of Leeds, Leeds, UK, [J.L.Meade@leeds.ac.uk](mailto:J.L.Meade@leeds.ac.uk)

**Prof Deirdre Ann Devine**, Division of Oral Biology, School of Dentistry, University of Leeds, Leeds, UK, [D.A.Devine@leeds.ac.uk](mailto:D.A.Devine@leeds.ac.uk)

**Prof Philip David Marsh**, Division of Oral Biology, School of Dentistry, University of Leeds, Leeds, UK, [p.d.marsh@leeds.ac.uk](mailto:p.d.marsh@leeds.ac.uk)


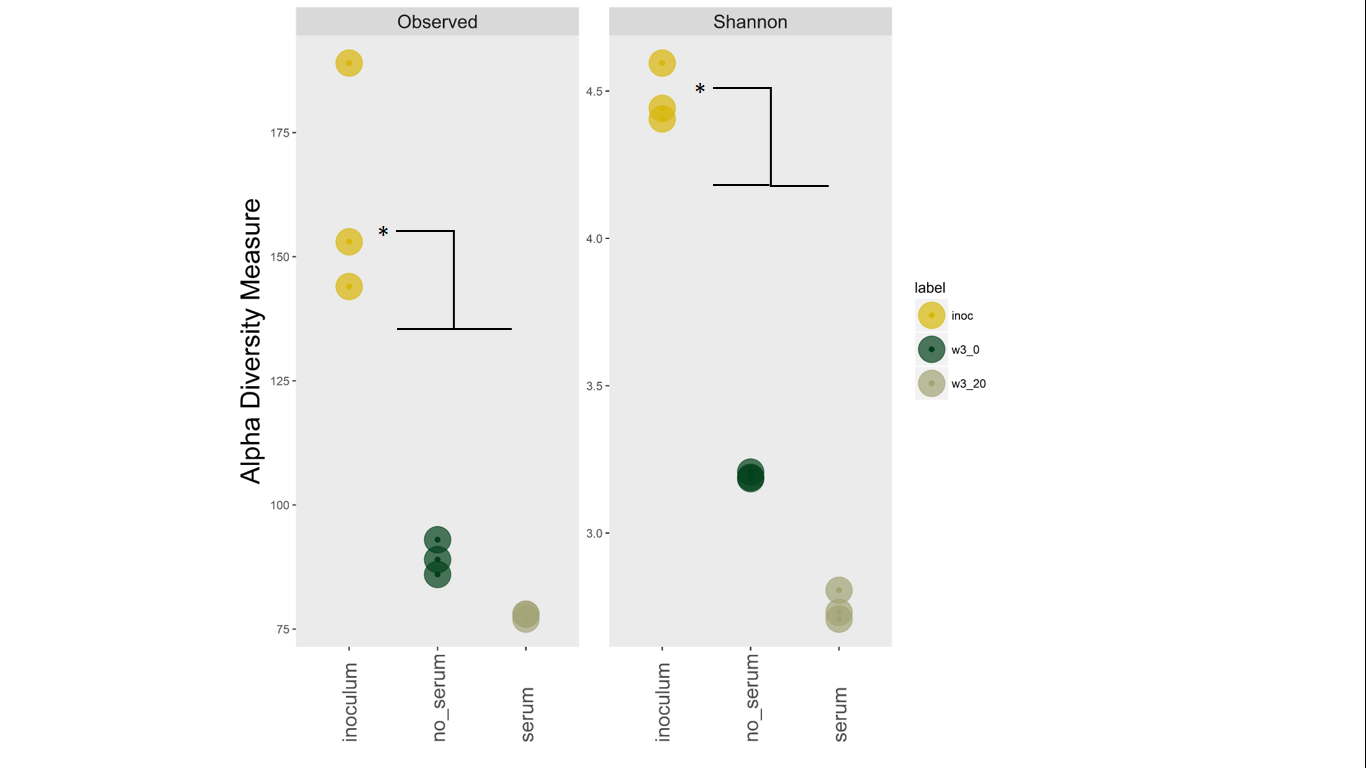


**Supplementary Figure 1**. **Alpha diversity measures of inoculum and biofilm samples**. Observed number of unique species and Shannon (richness and diversity) indexes were compared between inoculum and biofilms cultured in a protein-rich medium without (no serum) and with serum; * p<0.05, Tukey HSD test. Observed number of species was significantly higher for the inoculum (p < 0.05). The Shannon index, which describes community evenness by combining richness and diversity data, was also significantly higher for the inoculum (p < 0.05).


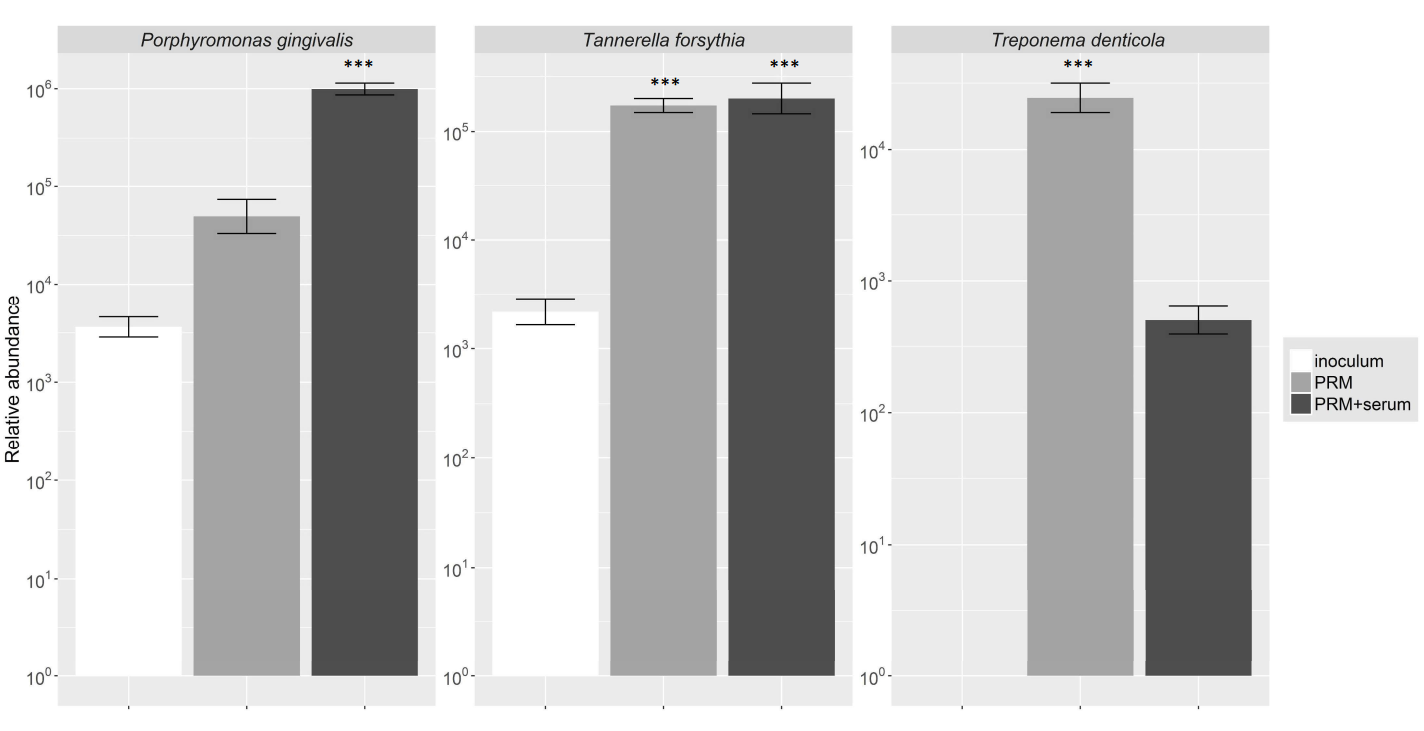


**Supplementary Figure 2.** **Difference in relative abundance of ‘red complex’ species following enrichment in protein-rich medium with or without supplementation with 20% (v/v) serum.** The graphs summarise the changes in relative abundance of metagenomes rarefied to the same sequencing depth between inoculum and biofilms following three weeks enrichment on a protein-rich medium with or without serum (the shotgun metagenomic data associated with *P. gingivalis*, *T. forsythia* and *T. denticola* were used to construct this figure). Asterisks mark the significant differences in relative abundance compared to the inoculum, *** - p < 0.001, HSD test. PRM – protein-rich medium.


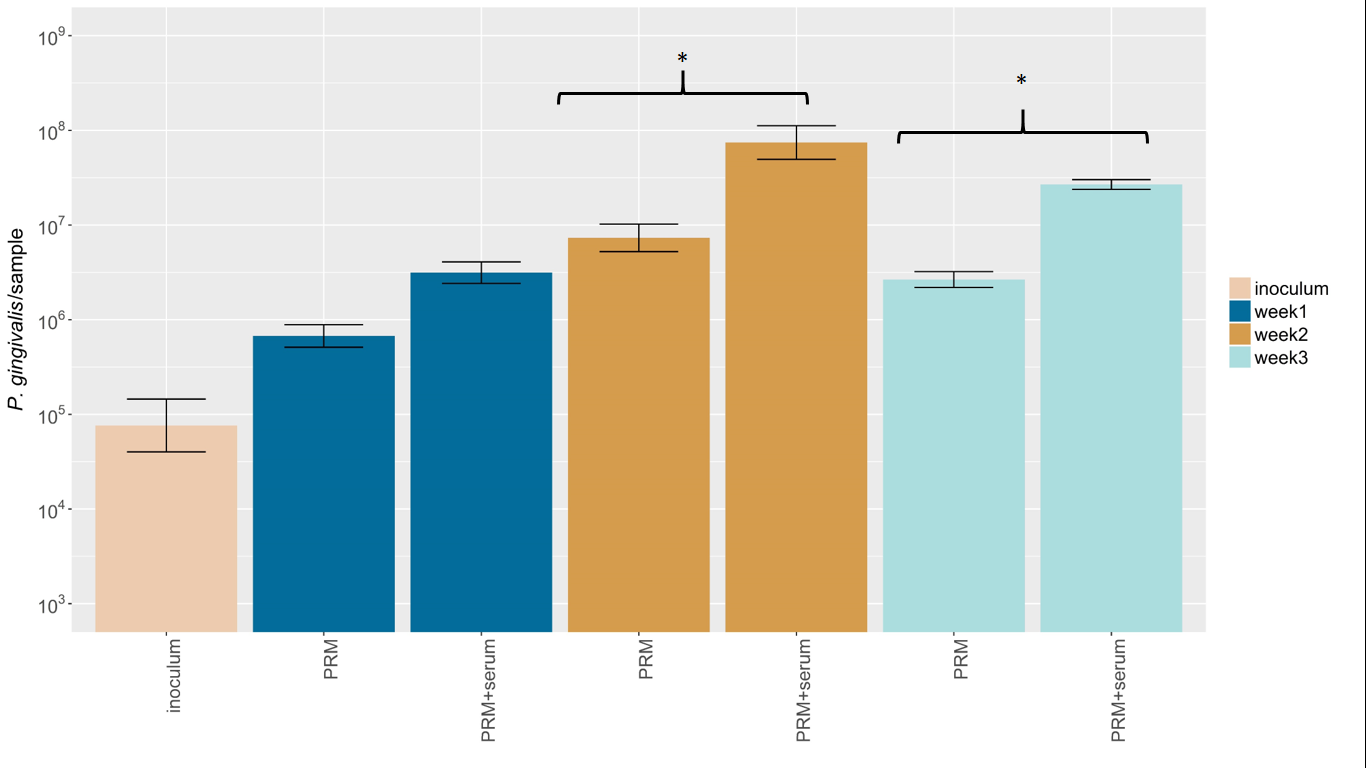


**Supplementary Figure 3.** **Abundance of *P. gingivalis* detected in inoculum and week one, week two and week three biofilms determined by qPCR**. In order to monitor *P. gingivalis* counts in the inoculum and week one – week three biofilms, a standard curve was generated for a range of concentrations of DNA, from 10 ng to 0.001 ng, in which the primers showed high linearity (R^2 = 0.99). Inoculum and biofilm samples were diluted to contain 10 ng of total DNA/reaction and were always in the linear range of the standard curve. The melting curve of PCR product showed that only one product was found. PRM – protein-rich medium, error bars represent standard deviation, * - p < 0.05, post hoc after Kruskal-Wallis test, n = 3 individual experiments, with 3 technical replicates each.

**Note on the methods used for the qPCR:**

Absolute counts of *P. gingivalis* were determined in each biofilm sample and inoculum using qPCR to confirm that the numbers of *P. gingivalis* changed throughout the experiment before undertaking sequencing. Primer pairs used in a study targeted *P. gingivalis* specific *waaA* gene encoding for lipid A synthesis (Hyvarinen et al., 2009). Serial dilutions of DNA from *P. gingivalis* W83 in nuclease free water was used at defined concentrations of 10 ng - 0.001 ng to generate a standard curve for qPCR reaction. For generation of a standard curve, qPCR was run in a total reaction volume of 10 µL, containing 5 µL of LightCycler® SYBR Green I Master mix (Roche Diagnostics Penzberg, Germany), 4 µL of DNA, 0.5 µL of 10mM primers. To quantify the numbers of *P. gingivalis* in the inoculum and biofilms, 4 µL containing 10 ng of DNA was used with 0.5 µL of 10mM primers and 5 µL of Master mix. Amplification was performed on a LightCycler 480 by initial denaturation for 5 min at 95°C, 40 cycles of 15 s at 95°C and 60 s at 60°C. Melting curve analysis was performed at 65°C for 1 min. *P. gingivalis* DNA concentration in each sample was calculated from the obtained crossing point (Cp) values and the abundance was calculated using the theoretical genome weight (Ammann et al., 2013).


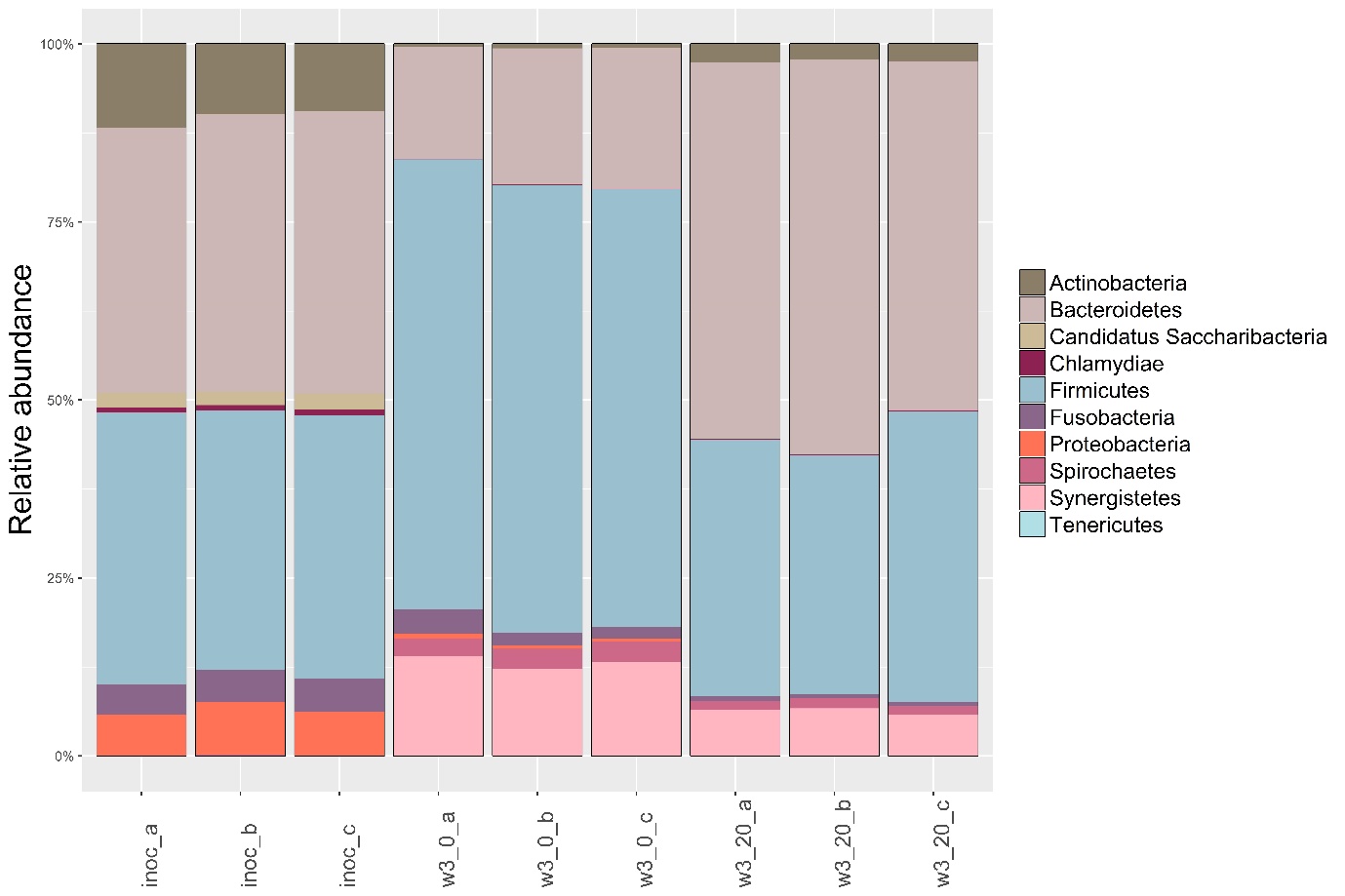


**Supplementary Figure 4.** **The taxonomic structure of the samples before and after enrichment.** Relative phyla distribution of the inoculum and of the biofilms following enrichment. Analysis based on the 16S rRNA data, extracted from the shotgun metagenomic data using the Human Oral Microbiome Database (HOMD) 16S rRNA database, shows the differences in relative phyla distribution between inoculum and biofilms enriched in a protein-rich medium ± serum. Inoc – inoculum sample, w3_0 – biofilms cultured in protein-rich medium, w3_20 – biofilm cultured in protein-rich medium with 20% serum (v/v), a, b, c – independent replicates.

**Supplementary Table 1**. **Species present in biofilms enriched following three weeks incubation** in a protein-rich medium supplemented with or without serum but which were not detected in the inoculum. The MEtaGenome ANalizer (MEGAN) was used to provide taxonomic annotations for reads using weighted lowest common ancestor (LCA) algorithm and recommended parameters. *Min-score* threshold was set to 80 and *top-percent* filter was set to 10%.

| **Detected in biofilms cultured in PRM** | **Detected in biofilms cultured in PRM + serum** | **Detected in biofilms enriched under both conditions** |
| --- | --- | --- |
| *Campylobacter rectus* | *Porphyromonadaceae* sp. H1 | *Eggerthia catenaformis* |
| *Colibacter massiliensis* | *Prevotella dentalis* | *Anaeroglobus geminatus* |
| *Desulfovibrio desulfuricans* | *Sphaerochaeta coccoides* | *Eubacterium nodatum* |
| *Fusobacterium* sp. CM21 | *Sphaerochaeta globosa* | *Phocaeicola abscessus* |
| *Bulleidia extructa* | *Porphyromonas gulae* | *Treponema denticola* |
| *Myroides odoratimimus* | *Actinomyces cardiffensis* | *Bacteroidetes* sp. OT 272 |
| *Capnocytophaga* sp. OT 338 | *Peptostreptococcaceae* sp. AS15 | *Peptostreptococcus anaerobius* CAG:621 |
| *Parabacteroides sp. Marseille*-P3160 | *Sphaerochaeta pleomorpha* | *Porphyromonadaceae* sp. COT-184 OH4590 |
| *Prevotella enoeca* |  | *Streptococcus constellatus* |
| *Streptococcus* sp. DD04 |  | *Pseudoramibacter alactolyticus* |
| *Anaerovorax odorimutans* |  | *Prevotella* sp. KH2C16 |
| *Streptococcus sinensis* |  | *Prevotella marshii* |
| *Aminobacterium mobile* |  | *Jonquetella anthropi* |
| *Dethiosulfovibrio salsuginis* |  | *Prevotella oralis* |
| uncultured *Clostridium* sp. |  | *Fusobacterium* sp. OT 370 |
| *Fusobacterium hwasookii* |  | *Slackia* sp. CM382 |
|  |  | *Prevotella buccae* |
|  |  | *Peptostreptococcaceae* sp. OT 113 |
|  |  | *Slackia exigua* |
|  |  | *Olsenella uli* |
|  |  | *Pyramidobacter* sp. C12-8 |
|  |  | *Alistipes* sp. CAG:435 |
|  |  | *Dialister invisus* |
|  |  | *Mogibacterium sp. CM50* |
|  |  | *Fusobacterium necrophorum* |
|  |  | *Mogibacterium timidum* |
|  |  | *Bacteroidales* sp. WCE2004 |
|  |  | *Treponema maltophilum* |
|  |  | *Alistipes* sp. CAG:514 |
|  |  | *Bacteroides* sp. CAG:709 |
|  |  | *Bacteroidales* sp. WCE2008 |
|  |  | *Dialister invisus* CAG:218 |
|  |  | *Parvimonas* sp. OT 110 |
|  |  | *Peptostreptococcus* sp. D1 |
|  |  | *Bacteroides* sp. CAG:1060 |
|  |  | *Anaerolineaceae* sp. OT 439 |
|  |  | *Erysipelotrichaceae* sp. NK3D112 |
|  |  | *Clostridioides difficile* |
|  |  | *Eubacterium yurii* |
|  |  | *Parvimonas* sp. OT 393 |

**Supplementary Table 2**. **The over-represented genes associated with functions**. Biofilms cultured in PRM with serum were compared against the inoculum with DESeq2 to see which genes coding for proteins were over-represented. Only functional groups with Log2FC > 1.0 are displayed.

| **Over-represented in PRM with serum** | **Fold**  **Change**  **(log2)** | **P adjusted** | **Over-represented in inoculum** | **Fold**  **Change (log2)** | **P adjusted** |
| --- | --- | --- | --- | --- | --- |
| Indole-pyruvate oxidoreductase complex | 5.17 | 2.7E-275 | SecY2-SecA2 Specialized Transport System | 6.92 | 2.2E-104 |
| Aromatic amino acid interconversions with aryl acids | 4.78 | 0 | Tellurite resistance: Chromosomal determinants | 6.80 | 1.5E-111 |
| Energy-conserving hydrogenase (ferredoxin) | 4.49 | 1.75E-41 | Biogenesis of cytochrome c oxidases | 6.64 | 4.17E-82 |
| Unknown sugar utilization (cluster yphABCDEFG) | 4.49 | 2.36E-27 | Glutathione-dependent pathway of formaldehyde detoxification | 6.39 | 4.54E-74 |
| CRISP Cmr Cluster | 4.31 | 3.4E-288 | Capsular Polysaccharide (CPS) of Campylobacter | 6.31 | 3.76E-77 |
| Group II intron-associated genes | 4.13 | 1.49E-84 | Glutathionylspermidine and Trypanothione | 6.29 | 2.1E-99 |
| A TRAP transporter and a hypothetical | 3.97 | 9.54E-28 | Ubiquinone Menaquinone-cytochrome c reductase complexes | 6.23 | 5.77E-96 |
| D-allose utilization | 3.66 | 1.48E-27 | Respiratory complex III (cytochrome b-c1) in plants | 6.22 | 7.41E-96 |
| Spore Core Dehydration | 3.62 | 4.42E-18 | ABC transporter of unknown substrate X | 6.17 | 1.27E-66 |
| Dimethylarginine metabolism | 3.58 | 8.3E-162 | Terminal cytochrome C oxidases | 5.99 | 2.2E-160 |
| Phosphoenolpyruvate phosphomutase | 3.42 | 1.25E-45 | Multidrug Resistance, Tripartite Systems Found in Gram Negative Bacteria | 5.94 | 6E-116 |
| Phosphonate metabolism | 3.42 | 1.25E-45 | Bacteriocin-like peptides Blp | 5.92 | 1.62E-50 |
| Propionyl-CoA to Succinyl-CoA Module | 3.13 | 8.8E-284 | Ferrous iron transporter EfeUOB, low-pH-induced | 5.83 | 1.3E-245 |
| Synechocystis experimental | 2.92 | 4.8E-156 | Phospholipid and Fatty acid biosynthesis related cluster | 5.76 | 9.9E-259 |
| H2:CoM-S-S-HTP oxidoreductase | 2.86 | 4.72E-10 | BOX, RUP and SPRITE repeats in Streptococcus pneumoniae | 5.72 | 1.43E-47 |
| Methanogenesis | 2.79 | 8.2E-10 | DNA repair, bacterial photolyase | 5.72 | 1.37E-46 |
| millsd methanogenesis | 2.79 | 8.2E-10 | Flavohaemoglobin | 5.58 | 2.8E-176 |
| Bacitracin Stress Response | 2.75 | 3.27E-11 | TN RidA: all subgroups, 981 genomes | 5.42 | 5.9E-179 |
| Sporulation gene orphans | 2.69 | 1.55E-12 | Biogenesis of cbb3-type cytochrome c oxidases | 5.37 | 1.07E-66 |
| Xylose utilization in plants | 2.60 | 2.62E-56 | Mercury resistance operon | 5.34 | 4.66E-68 |
| Diphthamide biosynthesis | 2.52 | 1.59E-71 | Petrobactin-mediated iron uptake system | 5.33 | 3.1E-38 |
| Cyanophycin Metabolism | 2.51 | 2.02E-70 | A conserved operon linked to TyrR and possibly involved in virulence | 5.33 | 3.35E-41 |
| Siderophore pyochelin | 2.47 | 4.02E-08 | p-Hydroxybenzoate degradation | 4.96 | 4.28E-34 |
| Putrescine utilization pathways | 2.45 | 7.93E-66 | Ergothioneine Cyano | 4.95 | 3.33E-38 |
| CoenzymeM Archaea | 2.42 | 1.42E-16 | Lipoprotein sorting system | 4.90 | 6.38E-33 |
| Unknown carbohydrate utilization ( cluster Yeg ) | 2.41 | 1.54E-78 | ESAT-6 proteins secretion system in Firmicutes | 4.87 | 3.3E-195 |
| Zinc resistance | 2.27 | 1.32E-26 | Glutamate transporter involved in acid tolerance in Streptococcus | 4.86 | 9.63E-32 |
| Aminoglycoside adenylyltransferases | 2.27 | 9.05E-14 | Phenylpropanoid compound degradation | 4.79 | 1.62E-33 |
| Lysine fermentation MCB 432 | 2.22 | 0 | Lactate Racemization | 4.77 | 1.19E-64 |
| Nicotinate catabolism, anaerobic | 2.21 | 3.29E-11 | Putative TldE-TldD proteolytic complex | 4.77 | 2.03E-34 |
| Tricarboxylate transport system | 2.13 | 1.62E-37 | ESAT-6 proteins secretion system in Mycobacteria (locus ESX-1) | 4.67 | 1.5E-128 |
| Lysine degradation | 2.10 | 0 | Cyanobacterial bypass in the TCA | 4.58 | 2.24E-33 |
| Xylose utilization | 2.08 | 3E-54 | Mycobacterium virulence operon involved in lipid degradation | 4.45 | 1.07E-24 |
| Malonate decarboxylase | 2.05 | 1.17E-05 | Fratricide in Streptococcus | 4.31 | 5.74E-87 |
| Malonate_ | 2.05 | 1.17E-05 | Characterized plastid transporters in plants | 4.05 | 2.72E-26 |
| Biofilm formation in Staphylococcus | 2.05 | 1.17E-22 | Respiratory complex IV (terminal cytochrome c oxidase) in plants | 3.98 | 2.43E-20 |
| Trehalose metabolism in plants | 2.02 | 2.33E-16 | Alkane synthesis in bacteria 2 | 3.87 | 3.02E-18 |
| Sphingolipid biosynthesis | 1.98 | 3.5E-168 | Head-to-head olefinic hydrocarbon biosynthesis | 3.87 | 3.02E-18 |
| Tetrathionate respiration | 1.96 | 3.37E-07 | Urease subunits | 3.84 | 1.3E-108 |
| Molybdopterin cytosine dinucleotide | 1.91 | 1.33E-05 | L-Cystine Uptake and Metabolism | 3.80 | 4.1E-280 |
| Benzoate degradation | 1.89 | 8.14E-05 | Iron(III) dicitrate transport system Fec | 3.69 | 6.82E-16 |
| Nitrogen fixation | 1.89 | 2.2E-12 | CBSS-452863.6.peg.1046 | 3.67 | 1.4E-102 |
| Isobutyryl-CoA to Propionyl-CoA Module | 1.87 | 8.2E-212 | Hexose Phosphate Uptake System | 3.62 | 3.16E-28 |
| Mediator of hyperadherence YidE in Enterobacteria and its conserved region | 1.83 | 0.000136 | Peptide ABC transport system Sap | 3.57 | 1.09E-19 |
| L-fucose utilization temp | 1.82 | 9.06E-61 | Orphan regulatory proteins | 3.50 | 7.94E-78 |
| Phage packaging machinery | 1.80 | 8.41E-28 | Formate dehydrogenase | 3.47 | 3.88E-47 |
| Polyhydroxybutyrate metabolism | 1.79 | 2.7E-174 | Proteasome archaeal | 3.47 | 1.87E-41 |
| Mycobacterium virulence operon involved in fatty acids biosynthesis | 1.78 | 3.35E-07 | 2-O-alpha-mannosyl-D-glycerate utilization | 3.42 | 8.1E-120 |
| Cannabinoid biosynthesis | 1.76 | 1.16E-49 | Acyl-CoA thioesterase II | 3.35 | 1.27E-24 |
| Chlorobenzoate degradation | 1.75 | 0.000309 | Isoprenoid biosynthesis in plants, mevalonate branch | 3.35 | 8.4E-250 |
| Beta-lactamase | 1.72 | 2.34E-05 | Urea decomposition | 3.26 | 1.6E-106 |
| V-Type ATP synthase | 1.71 | 0 | acinetobacter siderophore assembly | 3.20 | 3.8E-107 |
| V-Type ATP synthase in plants (vacuolar) | 1.71 | 0 | Siderophore assembly kit | 3.20 | 3.8E-107 |
| Sporulation draft | 1.69 | 0.000493 | Bilin Biosynthesis | 3.17 | 3.98E-12 |
| Two-component regulatory systems in Campylobacter | 1.65 | 4.54E-95 | Sodium Hydrogen Antiporter | 3.15 | 5.66E-27 |
| Na+ translocating decarboxylases and related biotin-dependent enzymes | 1.62 | 4.7E-136 | Purine nucleotide synthesis regulator | 3.00 | 1.14E-14 |
| Acetyl-CoA fermentation to Butyrate | 1.55 | 0 | Alkylphosphonate utilization | 2.98 | 6.81E-28 |
| SigmaB stress response regulation | 1.55 | 4.44E-31 | Late competence | 2.95 | 1.4E-122 |
| Rubrerythrin | 1.53 | 5.78E-61 | WhiB and WhiB-type regulatory proteins_ | 2.85 | 1.63E-10 |
| Unknown carbohydrate utilization (cluster Ydj ) | 1.50 | 0.002009 | Lipid-linked oligosaccharide synthesis related cluster | 2.83 | 4.66E-16 |
| Glutaconate CoA-transferase or 3-oxoadipate CoA-transferase Subunits | 1.47 | 8.05E-11 | CytR regulation | 2.78 | 8.22E-10 |
| ATP-dependent efflux pump transporter Ybh | 1.45 | 2.73E-09 | Polyglycerolphosphate lipoteichoic acid biosynthesis | 2.76 | 2.1E-124 |
| D-galactonate catabolism | 1.45 | 0.002872 | Gram positive Competence | 2.76 | 8.9E-133 |
| Pectin metabolism in plants | 1.45 | 1.14E-06 | Universal stress protein family | 2.75 | 3.57E-34 |
| Archease2 | 1.44 | 5.48E-05 | Glutathione analogs: mycothiol | 2.73 | 1.01E-08 |
| Resistance to Vancomycin | 1.43 | 2.92E-49 | Denitrification | 2.72 | 2.3E-106 |
| Cobalamin synthesis | 1.42 | 0 | trimethylamine N-oxide (TMAO) reductase | 2.70 | 4.15E-09 |
| Cob(I)alamin adenosyltransferase | 1.40 | 1.3E-33 | Tryptophan metabolism in plants | 2.62 | 0 |
| Heme biosynthesis orphans | 1.38 | 8.11E-06 | Iron Scavenging cluster in Thermus | 2.60 | 2.35E-09 |
| Lysine fermentation | 1.38 | 3.4E-243 | Pertussis toxin | 2.57 | 8.06E-08 |
| Conjugative transposon, Bacteroidales | 1.37 | 2.11E-82 | RNA 3'-terminal phosphate cyclase | 2.53 | 8.16E-30 |
| HMG CoA Synthesis | 1.33 | 3.21E-05 | Competence in Streptococci | 2.49 | 2.06E-76 |
| Isoprenoid scratch | 1.32 | 1.14E-16 | Streptococcus pyogenes virulence regulators | 2.48 | 1.46E-14 |
| Serotype determining Capsular polysaccharide biosynthesis in Staphylococcus | 1.32 | 0.001114 | HtrA and Sec secretion | 2.47 | 3.57E-19 |
| Bacterial Luciferases | 1.31 | 1.9E-99 | At1g26220 At1g32070 | 2.45 | 7.3E-08 |
| BactLuciferaseComplex | 1.31 | 1.9E-99 | Ethylmalonyl-CoA pathway of C2 assimilation, GJO | 2.44 | 1.26E-18 |
| NADH-dependent reduced ferredoxin:NADDP+ oxidoreductase | 1.29 | 1.1E-163 | Ethylmalonyl-CoA pathway of C2 assimilation | 2.44 | 1.36E-18 |
| Aromatic amino acid degradation | 1.24 | 1.23E-07 | FOL Commensurate regulon activation | 2.43 | 4.8E-195 |
| Flavodoxin | 1.22 | 2.36E-31 | Osmotic stress cluster | 2.42 | 2.31E-26 |
| Terminal cytochrome d ubiquinol oxidases | 1.21 | 3.62E-87 | Phosphoinositides biosynthesis in plants | 2.41 | 9.34E-13 |
| Catechol branch of beta-ketoadipate pathway | 1.21 | 1.13E-08 | Oxygen and light sensor PpaA-PpsR | 2.41 | 9.44E-08 |
| Terminal cytochrome oxidases | 1.21 | 1.78E-86 | Auxin biosynthesis | 2.39 | 9.7E-286 |
| B12 Cobalamin HMP | 1.19 | 0 | Dimethylsulfoniopropionate (DMSP) mineralization | 2.38 | 6.72E-07 |
| B12 Biosynthesis (Tavares copy1) | 1.19 | 0 | Osmoregulation | 2.37 | 7.3E-132 |
| Coenzyme B12 biosynthesis | 1.19 | 0 | Mycobacterial FadE proteins Acyl-CoA dehydrogenase | 2.36 | 9.25E-07 |
| Pyruvate:ferredoxin oxidoreductase | 1.18 | 1.38E-50 | Carotenoid biosynthesis | 2.35 | 5.92E-26 |
| An Arabinose Sensor | 1.17 | 5.1E-10 | D-Sorbitol(D-Glucitol) and L-Sorbose Utilization | 2.35 | 1.58E-23 |
| Na(+)-translocating NADH-quinone oxidoreductase and rnf-like group of electron transport complexes | 1.17 | 3.2E-115 | Extracellular Polysaccharide Biosynthesis of Streptococci | 2.32 | 3.39E-21 |
| Selenocysteine metabolism | 1.17 | 2.14E-74 | Prophage-encoded Exotoxins | 2.27 | 2.4E-07 |
| CRISPRs | 1.15 | 1.57E-79 | LOS core oligosaccharide biosynthesis | 2.26 | 2.1E-270 |
| Histidine Degradation in Streptococci | 1.15 | 7.6E-83 | Phage tail proteins 2 | 2.17 | 1.18E-11 |
| Nitrogen Fixation with NifL | 1.13 | 2.1E-112 | Methylcitrate cycle | 2.14 | 3.07E-63 |
| Histidine Degradation | 1.12 | 1.29E-58 | Streptococcus agalactiae hemolysin operon | 2.13 | 9.26E-06 |
| Lipid A-Ara4N pathway ( Polymyxin resistance ) | 1.12 | 2.53E-72 | Glutathione: Redox cycle | 2.12 | 6.9E-100 |
| Phage shock protein (psp) operon | 1.12 | 0.007282 | Type 4 conjugative transfer system, IncI1 type | 2.12 | 1.07E-05 |
| Mycobacterium virulence operon possibly involved in quinolinate biosynthesis | 1.12 | 3.04E-62 | Single-Rhodanese-domain proteins | 2.07 | 1.74E-05 |
| Biotin biosynthesis in plants | 1.11 | 6.28E-62 | Dehydrogenase complexes | 2.07 | 2.5E-184 |
| Flagellar motility | 1.09 | 2.65E-40 | Multidrug efflux pump in *Campylobacter jejuni* (CmeABC operon) | 2.06 | 5.9E-160 |
| Erythritol utilization | 1.09 | 0.02275 | Quinones biosynthesis HGM | 2.04 | 1.06E-84 |
| L-Arabinose CS | 1.08 | 2.94E-35 | rRNA modification Archaea | 2.02 | 6.11E-07 |
| Dimethylsulfoniopropionate (DMSP) mineralization, gjo | 1.07 | 1E-110 | Respiratory Complex I | 2.01 | 1.4E-281 |
| Dimethylsulfoniopropionate (DMSP) mineralization, WBW | 1.07 | 1E-110 | Menaquinone and Phylloquinone Biosynthesis | 1.98 | 1.75E-49 |
| T7-like cyanophage core proteins | 1.07 | 8.3E-36 | Broadly distributed proteins not in subsystems | 1.96 | 7.59E-30 |
| Polyprenyl Diphosphate Biosynthesis | 1.05 | 5.96E-27 | Quinone disambuiguation | 1.93 | 5.18E-76 |
| COG0523-Histidine | 1.05 | 7.73E-52 | Outer membrane | 1.92 | 5.67E-34 |
| Recycling of Peptidoglycan Amino Acids | 1.04 | 2.7E-119 | Sucrose utilization | 1.92 | 6.7E-207 |
| CBSS-565654.4.peg.3098 cAD1 cluster | 1.04 | 6.88E-84 | Phage tail fiber proteins | 1.90 | 4.71E-05 |
| L-Arabinose utilization | 1.04 | 9.38E-24 | Photosynthesis | 1.87 | 8.54E-13 |
| Anaerobic respiratory reductases | 1.04 | 4.6E-131 | Major Outer Membrane Proteins | 1.86 | 1.71E-28 |
| Chloroaromatic degradation pathway | 1.04 | 2.52E-06 | DNA repair, bacterial DinG and relatives | 1.82 | 1.78E-78 |
| Biotin HMP | 1.02 | 5.9E-68 | At1g48360 | 1.82 | 1.78E-78 |
| DNA topoisomerases, Type I, ATP-independent | 1.01 | 3.3E-268 | Menaquinone biosynthesis from chorismate via 1,4-dihydroxy-2-naphthoate | 1.80 | 3.85E-61 |
|  |  |  | tRNA splicing | 1.79 | 5.85E-10 |
|  |  |  | Tryptophan biosynthesis in Streptococci | 1.77 | 0 |
|  |  |  | Streptothricin resistance | 1.73 | 2.77E-05 |
|  |  |  | Steroid sulfates | 1.72 | 1.48E-62 |
|  |  |  | Coenzyme F420 hydrogenase | 1.72 | 1.55E-05 |
|  |  |  | Alpha-acetolactate operon | 1.68 | 1.57E-23 |
|  |  |  | Mercuric reductase | 1.63 | 1.15E-78 |
|  |  |  | Phylloquinone biosynthesis in plants | 1.60 | 2.61E-47 |
|  |  |  | Acetoin, butanediol metabolism | 1.59 | 1.1E-157 |
|  |  |  | Tagatose utilization | 1.59 | 9.9E-23 |
|  |  |  | Chorismate: Intermediate for synthesis of Tryptophan, PAPA antibiotics, PABA, 3-hydroxyanthranilate and more. | 1.59 | 1.4E-295 |
|  |  |  | Listeria Pathogenicity Island LIPI-1 extended | 1.58 | 6.44E-07 |
|  |  |  | Tryptophan synthesis | 1.58 | 9.5E-295 |
|  |  |  | AMP to 3-phosphoglycerate | 1.58 | 0.000809 |
|  |  |  | Siderophore Enterobactin | 1.57 | 0.001125 |
|  |  |  | Biflavanoid biosynthesis | 1.56 | 0.000414 |
|  |  |  | Flavonoids biosynthesis in plants | 1.56 | 0.000414 |
|  |  |  | Tannin biosynthesis | 1.56 | 0.000414 |
|  |  |  | Transport of Manganese | 1.56 | 3.08E-36 |
|  |  |  | Archaeal lipids | 1.54 | 1.9E-149 |
|  |  |  | Ketoisovalerate oxidoreductase | 1.52 | 2.49E-28 |
|  |  |  | Sucrose-specific PTS | 1.51 | 5.3E-141 |
|  |  |  | Control of cell elongation - division cycle in Bacilli | 1.49 | 7.9E-137 |
|  |  |  | Twin-arginine translocation system | 1.48 | 1.14E-16 |
|  |  |  | Mycobacterium virulence operon involved with a dormancy regulon | 1.48 | 1.08E-35 |
|  |  |  | Sucrose utilization Shewanella | 1.47 | 7.68E-57 |
|  |  |  | Exopolysaccharide Biosynthesis | 1.46 | 1.95E-21 |
|  |  |  | Fructose and Mannose Inducible PTS | 1.44 | 1.01E-72 |
|  |  |  | Gram-positive Extracellular Nucleases | 1.43 | 0.003243 |
|  |  |  | Cellulosome | 1.42 | 2.88E-61 |
|  |  |  | Glutathione: Biosynthesis and gamma-glutamyl cycle | 1.42 | 3.84E-68 |
|  |  |  | Regulation of Oxidative Stress Response | 1.36 | 9.16E-25 |
|  |  |  | Branched-chain amino acid metabolism in plants | 1.36 | 2.1E-215 |
|  |  |  | Khodge314 Isoleucine Biosynthesis | 1.36 | 2.9E-132 |
|  |  |  | Bacterial cyanide production and tolerance mechanisms | 1.35 | 2.52E-48 |
|  |  |  | FIG039061: hypothetical protein related to heme utilization | 1.34 | 7.04E-05 |
|  |  |  | Ubiquinone biosynthesis in plants | 1.32 | 4.85E-23 |
|  |  |  | Cadmium resistance | 1.32 | 4.65E-37 |
|  |  |  | ABC transporter tungstate (TC 3.A.1.6.2) | 1.30 | 0.006203 |
|  |  |  | Bacterial pathways for dimethylsulfoniopropionate and acrylate catabolism | 1.30 | 0.000284 |
|  |  |  | Pseudaminic Acid Biosynthesis | 1.30 | 0.001964 |
|  |  |  | RNA processing orphans | 1.29 | 0.002172 |
|  |  |  | Alpha-Amylase locus in *Streptocococcus* | 1.27 | 1.48E-22 |
|  |  |  | Uptake of selenate and selenite | 1.26 | 1.1E-06 |
|  |  |  | Biosynthesis of Arabinogalactan in Mycobacteria | 1.25 | 1.16E-23 |
|  |  |  | Soluble electron carriers in plants | 1.24 | 0.006492 |
|  |  |  | *Streptococcus pyogenes* Virulome | 1.21 | 3.35E-21 |
|  |  |  | Phage integration and excision | 1.21 | 6.72E-05 |
|  |  |  | Branched-Chain Amino Acid Biosynthesis | 1.20 | 3.8E-156 |
|  |  |  | Ubiquinone Biosynthesis | 1.20 | 9.33E-48 |
|  |  |  | Ubiquinone biosynthesis -- gjo | 1.20 | 9.33E-48 |
|  |  |  | Nitrate and nitrite ammonification | 1.19 | 3.6E-107 |
|  |  |  | SLO-NADGH Locus | 1.18 | 0.009017 |
|  |  |  | Phage entry and exit | 1.17 | 0.009357 |
|  |  |  | 2-phosphoglycolate salvage | 1.14 | 1.52E-16 |
|  |  |  | Galactose-inducible PTS | 1.13 | 5.16E-44 |
|  |  |  | Sulfur oxidation | 1.13 | 2.16E-06 |
|  |  |  | Muconate lactonizing enzyme family | 1.10 | 3.23E-12 |
|  |  |  | RidA5 subgroup | 1.09 | 2.28E-20 |
|  |  |  | Protection from Reactive Oxygen Species | 1.07 | 6.96E-53 |
|  |  |  | NiFe hydrogenase maturation | 1.07 | 3.12E-31 |
|  |  |  | SpeB-SpeF extended regulon | 1.06 | 0.027527 |
|  |  |  | EC 4.1.3.- Oxo-acid-lyases | 1.05 | 3.11E-89 |
|  |  |  | O-Methyl Phosphoramidate Capsule Modification in Campylobacter | 1.05 | 0.032656 |
|  |  |  | RpoS Regulators SG1 | 1.04 | 0.02745 |
|  |  |  | Choline and Betaine Uptake and Betaine Biosynthesis | 1.02 | 3.45E-09 |
|  |  |  | Creatine and Creatinine Degradation | 1.02 | 2.26E-09 |
|  |  |  | Quinones HGM | 1.01 | 1.77E-47 |
|  |  |  | Mannitol Utilization | 1.00 | 4.29E-59 |

**Supplementary Table 3**. **The over-represented genes associated with functions**. Biofilms cultured in PRM were compared against the inoculum with DESeq2 to see which genes coding for proteins were over-represented. Only functional groups with Log2FC > 1.0 are displayed.

| **Over-represented in PRM** | **Fold Change log2** | **Over-represented in inoculum** | **Fold Change log2** |
| --- | --- | --- | --- |
| Energy-conserving hydrogenase (ferredoxin) | 5.1*** | SecY2-SecA2 Specialized Transport System | 7.0*** |
| Unknown sugar utilization (cluster yphABCDEFG) | 5.1*** | MukBEF Chromosome Condensation | 6.6*** |
| H2:CoM-S-S-HTP oxidoreductase | 4.6*** | Glutathione-dependent pathway of formaldehyde detoxification | 6.5*** |
| Methanogenesis | 4.5*** | TN RidA: all subgroups, 981 genomes | 6.1*** |
| millsd methanogenesis | 4.5*** | ABC transporter of unknown substrate X | 5.9*** |
| Indole-pyruvate oxidoreductase complex | 3.8*** | Bacteriocin-like peptides Blp | 5.8*** |
| Aromatic amino acid interconversions with aryl acids | 3.8*** | BOX, RUP and SPRITE repeats in *Streptococcus pneumoniae* | 5.7*** |
| Chlorobenzoate degradation | 3.8*** | Mercury resistance operon | 5.6*** |
| A TRAP transporter and a hypothetical | 3.6*** | Biogenesis of cytochrome c oxidases | 5.5*** |
| Phosphoenolpyruvate phosphomutase | 3.6*** | DNA repair, bacterial photolyase | 5.5*** |
| Phosphonate metabolism | 3.6*** | Capsular Polysaccharide (CPS) of *Campylobacter* | 5.4*** |
| D-galactonate catabolism | 3.4*** | Urease subunits | 5.1*** |
| Group II intron-associated genes | 3.2*** | A conserved operon linked to TyrR and possibly involved in virulence | 5.1*** |
| Sulfate reduction-associated complexes | 2.9*** | Petrobactin-mediated iron uptake system | 5.1*** |
| Tricarboxylate transport system | 2.9*** | Ergothioneine Cyano | 5.0*** |
| Spore Core Dehydration | 2.9*** | Fratricide in *Streptococcus* | 4.9*** |
| Mycobacterium virulence operon involved in fatty acids biosynthesis | 2.9*** | CBSS-452863.6.peg.1046 | 4.8*** |
| Diphthamide biosynthesis | 2.7*** | Glutamate transporter involved in acid tolerance in *Streptococcus* | 4.8*** |
| Cyanophycin Metabolism | 2.7*** | Lipoprotein sorting system | 4.8*** |
| Nicotinate catabolism, anaerobic | 2.7*** | Glutathionylspermidine and Trypanothione | 4.8*** |
| ATP-dependent efflux pump transporter Ybh | 2.7*** | Proteasome archaeal | 4.7*** |
| Serotype determining Capsular polysaccharide biosynthesis in Staphylococcus | 2.6*** | Phospholipid and Fatty acid biosynthesis related cluster | 4.7*** |
| CRISP Cmr Cluster | 2.6*** | Terminal cytochrome C oxidases | 4.6*** |
| Archease2 | 2.5*** | p-Hydroxybenzoate degradation | 4.6*** |
| D-allose utilization | 2.5*** | *Mycobacterium* virulence operon involved in lipid degradation | 4.4*** |
| Aromatic amino acid degradation | 2.4*** | Phenylpropanoid compound degradation | 4.4*** |
| HMG CoA Synthesis | 2.4*** | Ubiquinone Menaquinone-cytochrome c reductase complexes | 4.4*** |
| Xylose utilization | 2.4*** | Biogenesis of cbb3-type cytochrome c oxidases | 4.3*** |
| Tetrathionate respiration | 2.4*** | Acyl-CoA thioesterase II | 4.2*** |
| Sporulation gene orphans | 2.4*** | Sodium Hydrogen Antiporter | 4.2*** |
| Two-component regulatory systems in Campylobacter | 2.4*** | Cyanobacterial bypass in the TCA | 4.1*** |
| Putrescine utilization pathways | 2.3*** | Ferrous iron transporter EfeUOB, low-pH-induced | 4.0*** |
| Heme biosynthesis orphans | 2.3*** | Urea decomposition | 3.9*** |
| Zinc resistance | 2.2*** | Alkane synthesis in bacteria 2 | 3.9*** |
| Propionyl-CoA to Succinyl-CoA Module | 2.2*** | Head-to-head olefinic hydrocarbon biosynthesis | 3.9*** |
| IbrA and IbrB: co-activators of prophage gene expression | 2.1*** | Flavohaemoglobin | 3.9*** |
| Bacitracin Stress Response | 2.1*** | CytR regulation | 3.9*** |
| Beta-lactamase | 2.1*** | Peptide ABC transport system Sap | 3.8*** |
| Glutaconate CoA-transferase or 3-oxoadipate CoA-transferase Subunits | 2.0*** | Lipid-linked oligosaccharide synthesis related cluster | 3.8*** |
| Isobutyryl-CoA to Propionyl-CoA Module | 2.0*** | Lactate Racemization | 3.7*** |
| Biofilm formation in Staphylococcus | 2.0*** | Iron(III) dicitrate transport system Fec | 3.6*** |
| Catechol branch of beta-ketoadipate pathway | 2.0*** | Biflavanoid biosynthesis | 3.6*** |
| Heme and heme d1 biosynthesis from siroheme | 2.0*** | Tannin biosynthesis | 3.6*** |
| Cannabinoid biosynthesis | 1.9*** | Tellurite resistance: Chromosomal determinants | 3.5*** |
| Integrons | 1.9*** | Hexose Phosphate Uptake System | 3.5*** |
| Molybdopterin cytosine dinucleotide | 1.8*** | RpoS Regulators SG1 | 3.4*** |
| RidA: all subgroups, 8100 genomes | 1.8*** | 4-Hydroxyphenylacetic acid catabolic pathway | 3.4*** |
| Flagellar motility | 1.8*** | Bilin Biosynthesis | 3.3*** |
| Nitrogen fixation | 1.8*** | Cyanate hydrolysis | 3.1*** |
| Siderophore pyochelin | 1.7*** | Oxygen and light sensor PpaA-PpsR | 3.1*** |
| Lysine fermentation MCB 432 | 1.7*** | At1g26220 At1g32070 | 3.0*** |
| An Arabinose Sensor | 1.7*** | Multidrug Resistance, Tripartite Systems Found in Gram negative Bacteria | 3.0*** |
| Aminoglycoside adenylyltransferases | 1.7*** | Phage tail proteins 2 | 2.9*** |
| Unknown carbohydrate utilization (cluster Yeg ) | 1.7*** | WhiB and WhiB-type regulatory proteins_ | 2.9*** |
| L-Arabinose utilization | 1.7*** | Methylcitrate cycle | 2.9*** |
| Carbon monoxide induced hydrogenase | 1.7*** | rRNA modification Archaea | 2.8*** |
| SigmaB stress response regulation | 1.6*** | Broadly distributed proteins not in subsystems | 2.8*** |
| Bacterial Chemotaxis | 1.6*** | Orphan regulatory proteins | 2.8*** |
| EC 3.4.19.- Omega peptidases | 1.6*** | Extracellular Polysaccharide Biosynthesis of Streptococci | 2.7*** |
| Lipid A-Ara4N pathway (Polymyxin resistance ) | 1.6*** | Bacterial cyanide production and tolerance mechanisms | 2.6*** |
| L-Arabinose CS | 1.6*** | Capsular Polysaccharides Biosynthesis and Assembly | 2.5*** |
| Polyhydroxybutyrate metabolism | 1.6*** | Ethylmalonyl-CoA pathway of C2 assimilation | 2.5*** |
| Chloroaromatic degradation pathway | 1.5*** | ESAT-6 proteins secretion system in Firmicutes | 2.5*** |
| Sporulation draft | 1.5** | Ethylmalonyl-CoA pathway of C2 assimilation, GJO | 2.5*** |
| Carnitine Metabolism in Microorganisms | 1.5** | L-Cystine Uptake and Metabolism | 2.5*** |
| Malonate decarboxylase | 1.5** | Quinone oxidoreductase family | 2.5*** |
| Malonate_ | 1.5** | Alkylphosphonate utilization | 2.4*** |
| D-gluconate and ketogluconates metabolism | 1.5*** | Respiratory Complex I | 2.4*** |
| L-fucose utilization temp | 1.4*** | Universal stress protein family | 2.4*** |
| Isoprenoid scratch | 1.4*** | trimethylamine N-oxide (TMAO) reductase | 2.4*** |
| p-Aminobenzoyl-Glutamate Utilization | 1.4*** | Purine nucleotide synthesis regulator | 2.3*** |
| D-Galacturonate and D-Glucuronate Utilization | 1.4*** | beta carboxysome | 2.3*** |
| V-Type ATP synthase | 1.4*** | Mycobacterial FadE proteins Acyl-CoA dehydrogenase | 2.3*** |
| Phage packaging machinery | 1.4*** | CO2 uptake, carboxysome | 2.3*** |
| Lysine degradation | 1.4*** | Dimethylsulfoniopropionate (DMSP) mineralization | 2.3*** |
| Flagellum | 1.4*** | Streptococcus agalactiae hemolysin operon | 2.2*** |
| Bacterial hemoglobins | 1.3*** | Phage tail fiber proteins | 2.2*** |
| MazEF toxin-antitoxing (programmed cell death) system | 1.3*** | PII superfamily | 2.2*** |
| Pyruvate:ferredoxin oxidoreductase | 1.3*** | ESAT-6 proteins secretion system in Mycobacteria (locus ESX-1) | 2.2*** |
| Acetyl-CoA fermentation to Butyrate | 1.3*** | D-Sorbitol(D-Glucitol) and L-Sorbose Utilization | 2.1*** |
| Flavodoxin | 1.3*** | Single-Rhodanese-domain proteins | 2.1*** |
| Rubrerythrin | 1.3*** | Type 4 conjugative transfer system, IncI1 type | 2.1*** |
| Terminal cytochrome d ubiquinol oxidases | 1.2*** | HtrA and Sec secretion | 2.1*** |
| Terminal cytochrome oxidases | 1.2*** | Osmotic stress cluster | 2.0*** |
| Dimethylarginine metabolism | 1.2*** | Prophage-encoded Exotoxins | 2.0*** |
| beta-glucuronide utilization | 1.2*** | Dehydrogenase complexes | 1.9*** |
| Anaerobic respiratory reductases | 1.2*** | 2-O-alpha-mannosyl-D-glycerate utilization | 1.9*** |
| Teichuronic acid biosynthesis | 1.2*** | Late competence | 1.9*** |
| Taurine Utilization | 1.2* | Aromatic Amine Catabolism | 1.8*** |
| CBSS-565654.4.peg.3098 cAD1 cluster | 1.1*** | Denitrification | 1.8*** |
| Benzoate catabolism | 1.1*** | Cellulosome | 1.8*** |
| Citrate Metabolism KE2 | 1.1*** | Menaquinone and Phylloquinone Biosynthesis | 1.8*** |
| Na+ translocating decarboxylases and related biotin-dependent enzymes | 1.1*** | Quinones biosynthesis HGM | 1.8*** |
| Nitrogen Fixation with NifL | 1.1*** | Polyglycerolphosphate lipoteichoic acid biosynthesis | 1.7*** |
| Streptococcal Hyaluronic Acid Capsule | 1.1*** | Menaquinone biosynthesis from chorismate via 1,4-dihydroxy-2-naphthoate | 1.7*** |
| Histidine Degradation | 1.1*** | Gram positive Competence | 1.7*** |
| ECF class transporters | 1.1*** | Tryptophan biosynthesis in streptococci | 1.7*** |
| Histidine Degradation in Streptococci | 1.0*** | Glutathione: Redox cycle | 1.7*** |
| SCIFF peptide maturase system | 1.0*** | *Listeria* Pathogenicity Island LIPI-1 extended | 1.7*** |
| Dimethylsulfoniopropionate (DMSP) mineralization, gjo | 1.0*** | Auxin biosynthesis | 1.7*** |
| Dimethylsulfoniopropionate (DMSP) mineralization, WBW | 1.0*** | Ketoisovalerate oxidoreductase | 1.6*** |
| NADH-dependent reduced ferredoxin:NADDP+ oxidoreductase | 1.0*** | Acinetobacter siderophore assembly | 1.6*** |
| Lysine fermentation | 1.0*** | Siderophore assembly kit | 1.6*** |
| Cobalamin synthesis | 1.0*** | RNA processing orphans | 1.6*** |
| Transport of Nickel and Cobalt | 1.0*** | archaeal lipids bobik | 1.6*** |
| COG0523-Histidine | 1.0*** | AMP to 3-phosphoglycerate | 1.6*** |
| Toxin-antitoxin replicon stabilization systems | 1.0* | Formate dehydrogenase | 1.6*** |
| Cyanobacterial Circadian Clock | 1.0*** | Osmoregulation | 1.6*** |
| The mdtABCD multidrug resistance cluster | 1.0* | Streptothricin resistance | 1.6*** |
|  |  | Archaeal lipids | 1.5*** |
|  |  | Exopolysaccharide Biosynthesis | 1.5*** |
|  |  | Tryptophan synthesis | 1.4*** |
|  |  | Chorismate: Intermediate for synthesis of Tryptophan, PAPA antibiotics, PABA, 3-hydroxyanthranilate and more. | 1.4*** |
|  |  | Control of cell elongation - division cycle in bacilli | 1.4*** |
|  |  | Xanthine Metabolism in Bacteria | 1.4*** |
|  |  | Competence in streptococci | 1.3*** |
|  |  | DNA repair, bacterial DinG and relatives | 1.3*** |
|  |  | At1g48360 | 1.3*** |
|  |  | Protection from Reactive Oxygen Species | 1.3*** |
|  |  | Putative TldE-TldD proteolytic complex | 1.3*** |
|  |  | RNA 3'-terminal phosphate cyclase | 1.3*** |
|  |  | Mercuric reductase | 1.3*** |
|  |  | Transport of manganese | 1.3*** |
|  |  | Nitrate and nitrite ammonification | 1.2*** |
|  |  | FIG039061: hypothetical protein related to heme utilization | 1.2*** |
|  |  | LOS core oligosaccharide biosynthesis | 1.2*** |
|  |  | Sulfur oxidation | 1.2*** |
|  |  | Glutathione analogs: mycothiol | 1.2** |
|  |  | Coenzyme F420 hydrogenase | 1.2** |
|  |  | Phage entry and exit | 1.2* |
|  |  | Alkaloid biosynthesis from L-lysine | 1.2* |
|  |  | Glutaredoxins | 1.1*** |
|  |  | Outer membrane | 1.1*** |
|  |  | Quinones HGM | 1.1*** |
|  |  | Major Outer Membrane Proteins | 1.1*** |
|  |  | Carotenoid biosynthesis | 1.1*** |
|  |  | Sucrose utilization | 1.1*** |
|  |  | Transport of Zinc | 1.1*** |
|  |  | Tagatose utilization | 1.1*** |
|  |  | Lactate utilization temp | 1.1*** |
|  |  | Siderophore Enterobactin | 1.1* |
|  |  | SpeB-SpeF extended regulon | 1.1* |
|  |  | Autoinducer 2 (AI-2) transport and processing (lsrACDBFGE operon) | 1.0*** |
|  |  | *Streptococcus pyogenes* virulence regulators | 1.0*** |
|  |  | Quinone disambuiguation | 1.0*** |
|  |  | FOL Commensurate regulon activation | 1.0*** |
|  |  | Steroid sulfates | 1.0*** |
|  |  | Phage head and packaging | 1.0** |
|  |  | Gram-positive Extracellular Nucleases | 1.0* |

**Supplementary Table 4. Summary of over-represented genes associated with functions in inoculum and biofilms enriched in protein-rich medium (PRM) with and without supplementation of serum.** Samples of the inoculum were compared with biofilms cultured in PRM with and without serum with DESeq2 to see which genes coding for proteins were over-represented. * - p < 0.05, ** - p < 0.01 *** - p < 0.001, Wald test.

| **Comparison between inoculum and biofilms in PRM** | | | |
| --- | --- | --- | --- |
| **Over-represented in inoculum** | | **Over-represented in PRM** | |
| **Functional group** | **Fold change (log2)** | **Functional group** | **Fold change (log2)** |
| Secondary Metabolism | 0.96*** | Arabinose Sensor and transport module | 0.76*** |
| Regulation and Cell signalling | 0.28*** | Motility and Chemotaxis | 0.44*** |
| General Stress Response and Stationary Phase Response | 0.27*** | Nucleotide sugars | 0.33*** |
| Potassium metabolism | 0.26** | Metabolism of Aromatic Compounds | 0.25*** |
| Nitrogen Metabolism | 0.18*** | Phosphorus Metabolism | 0.22*** |
| Sulphur Metabolism | 0.16*** | Fatty Acids, Lipids, and Isoprenoids | 0.21*** |
| Stress Response | 0.14*** | Respiration | 0.12*** |
| Cell Wall and Capsule | 0.13*** | Protein Metabolism | 0.08*** |
| Cell Division and Cell Cycle | 0.11*** |  |  |
| **Comparison between inoculum and biofilms in PRM with serum** | | | |
| **Over-represented in inoculum** | | **Over-represented in PRM with serum** | |
| **Functional group** | **Fold Change (log2)** | **Functional group** | **Fold Change (log2)** |
| General Stress Response and Stationary Phase Response | 1.97** | Arabinose Sensor and transport module | 1.19*** |
| Secondary Metabolism | 1.35*** | Phages, Prophages, Transposable elements, Plasmids | 0.55*** |
| Regulation and Cell signalling | 0.39*** | Phosphorus Metabolism | 0.38*** |
| Central metabolism | 0.21** | Metabolism of Aromatic Compounds | 0.34*** |
| Cell Wall and Capsule | 0.18*** | Nucleotide sugars | 0.27*** |
| Membrane Transport | 0.18*** | Polyamines | 0.26*** |
| Potassium metabolism | 0.17*** | Motility and Chemotaxis | 0.22*** |
| Nitrogen Metabolism | 0.15*** | Transcriptional regulation | 0.19*** |
| Sulphur Metabolism | 0.15*** | Thiamin | 0.18*** |
| Stress Response | 0.14*** | Fatty Acids, Lipids, and Isoprenoids | 0.17*** |
| Metabolite damage and its repair or mitigation | 0.13*** | Cofactors, Vitamins, Prosthetic Groups, Pigments | 0.11*** |
| Cell Division and Cell Cycle | 0.11*** | Respiration | 0.11*** |
| Carbohydrates | 0.09*** | Protein Metabolism | 0.10*** |
|  |  | Dormancy and Sporulation | 0.09*** |
|  |  | Miscellaneous | 0.08*** |
|  |  | DNA Metabolism | 0.06*** |
|  |  | Virulence | 0.03*** |
| **Comparison between biofilms cultured in PRM and PRM with serum** | | | |
| **Over-represented in PRM** | | **Over-represented in PRM with serum** | |
| **Functional group** | **Fold Change (log2)** | **Functional group** | **Fold Change (log2)** |
| Central metabolism | 0.47*** | Phages, Prophages, Transposable elements, Plasmids | 0.46*** |
| Secondary Metabolism | 0.39*** | Motility and Chemotaxis | 0.4*** |
| Membrane Transport | 0.33*** | Thiamin | 0.28*** |
| Arabinose Sensor and transport module | 0.32*** | Polyamines | 0.27* |
| Metabolite damage and its repair or mitigation | 0.22*** | Phages, Prophages, Transposable elements | 0.25*** |
| Nucleotide sugars | 0.16*** | Iron acquisition and metabolism | 0.21*** |
| Unclassified | 0.12*** | Phosphorus Metabolism | 0.18*** |
| Regulation and Cell signalling | 0.11** | Virulence, Disease and Defense | 0.16*** |
| Amino Acids and Derivatives | 0.09** | Metabolism of Aromatic Compounds | 0.14** |
| Cell Wall and Capsule | 0.08*** | Transcriptional regulation | 0.12** |
| Carbohydrates | 0.08** | Cofactors, Vitamins, Prosthetic Groups, Pigments | 0.1*** |
|  |  | Nucleosides and Nucleotides | 0.08** |
|  |  | DNA Metabolism | 0.06*** |

**Supplementary Dataset: List of genes and corresponding read count for all biofilm sample replicates.** The read count has been normalized using the R package DESeq2, which uses a negative binomial distribution algorithm.
